# Supplementary figures and images for: A major role of class III HD-ZIPs in promoting sugar beet cyst nematode parasitism in Arabidopsis
Source: PLoS Pathog. 2024 Nov 7;20(11):e1012610. doi: 10.1371/journal.ppat.1012610 (PMC11542791; doi:10.1371/journal.ppat.1012610)

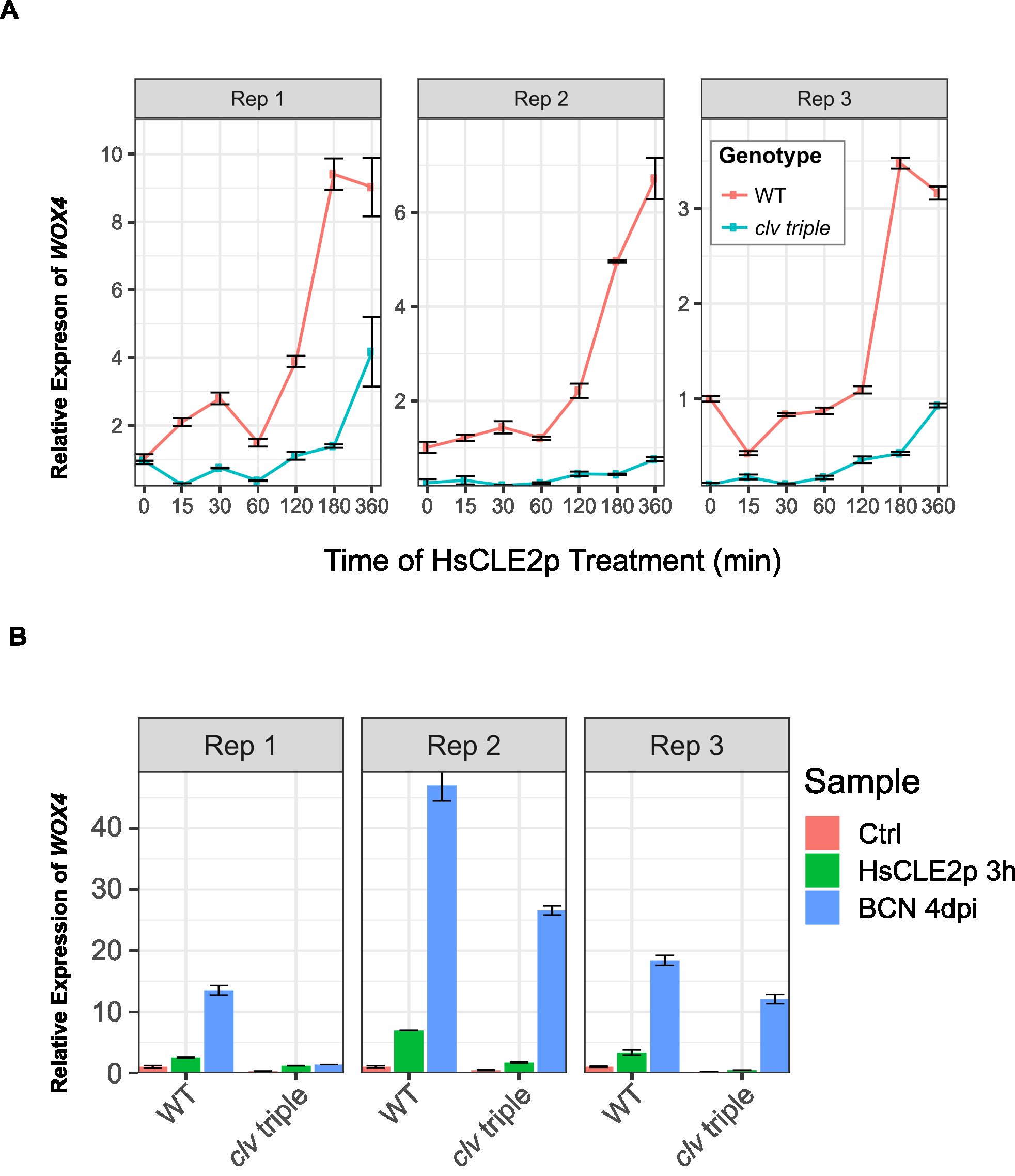

Supplement: S1 Fig — A. Response of WOX4 gene expression to HsCLE2p treatment was quantified by qPCR to find an optimal time point for HsCLE2p treatment. The 180 min was selected as the treatment time for RNAseq samples. B. Induction of WOX4 gene in RNA sequencing samples by qPCR. (TIF) [file ppat.1012610.s001.tif]

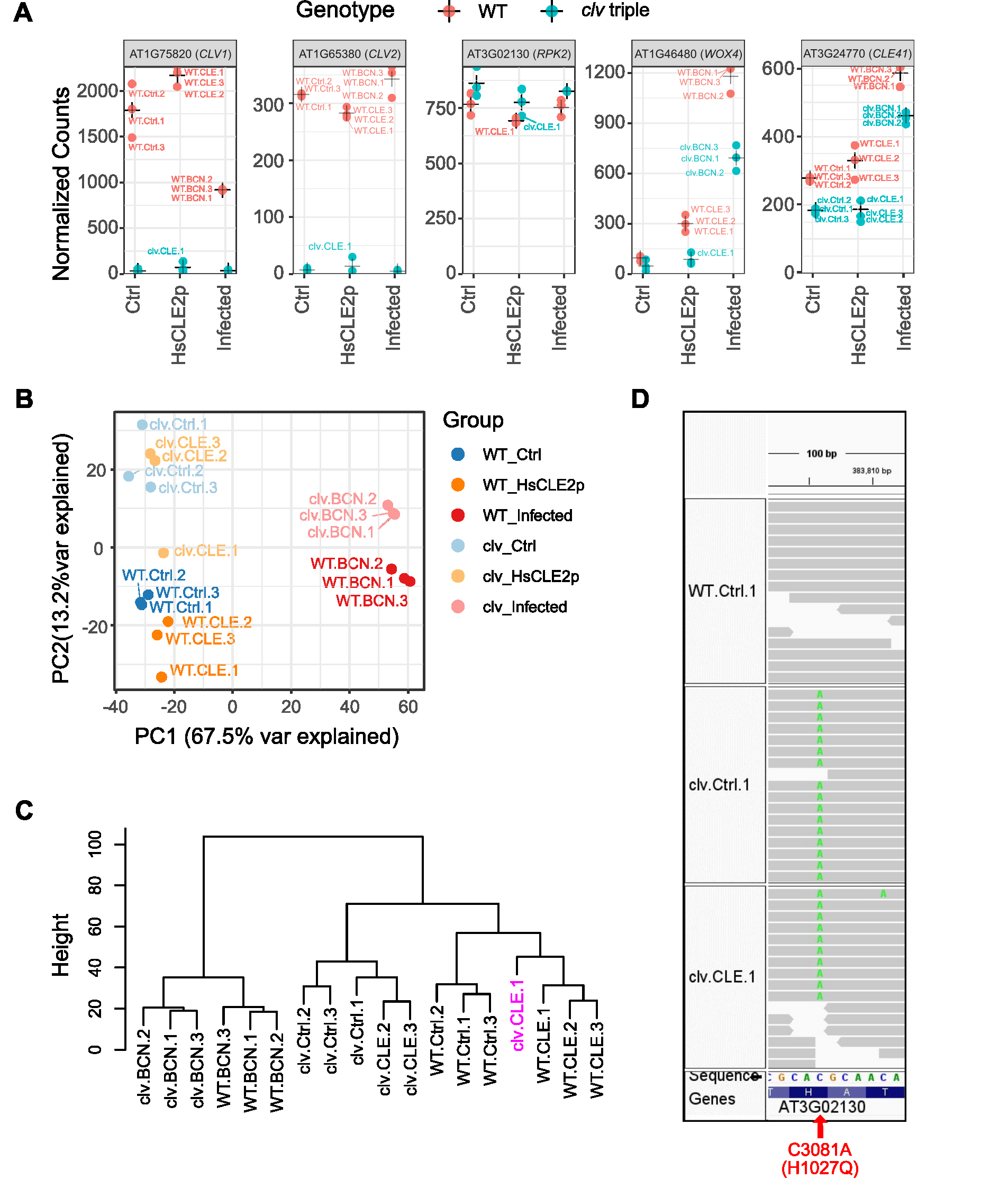

Supplement: S2 Fig — A. Expression of CLV1, CLV2, RPK2, WOX4, and CLE41 genes in the RNAseq dataset. Expression of CLV1 and CLV2 are barely detectable in the clv triple mutant due to clv1-101 and clv2-101 being null mutants. RPK2 gene expression is comparable in the clv triple mutant and wild-type due to the rpk2-5 allele being a point mutation (C3081A). B. PCA plot of RNAseq samples. All samples clustered as expected except for the “clv.CLE.1” sample, which was removed from subsequent differential gene expression analysis. C. Hierarchical clustering of RNAseq samples. Sample clv.CLE.1 (magenta) clustered with wild-type samples. The cluster is constructed using Euclidean distance with the ‘complete’ agglomeration method. D. The clv triple mutant showing the C3081A mutation (H1027Q on amino acid level) at the RPK2 locus (AT3G02130) on the RNA-seq reads. Reads alignment was visualized using Integrated Genome Viewer (v2.11.3). (TIF) [file ppat.1012610.s002.tif]

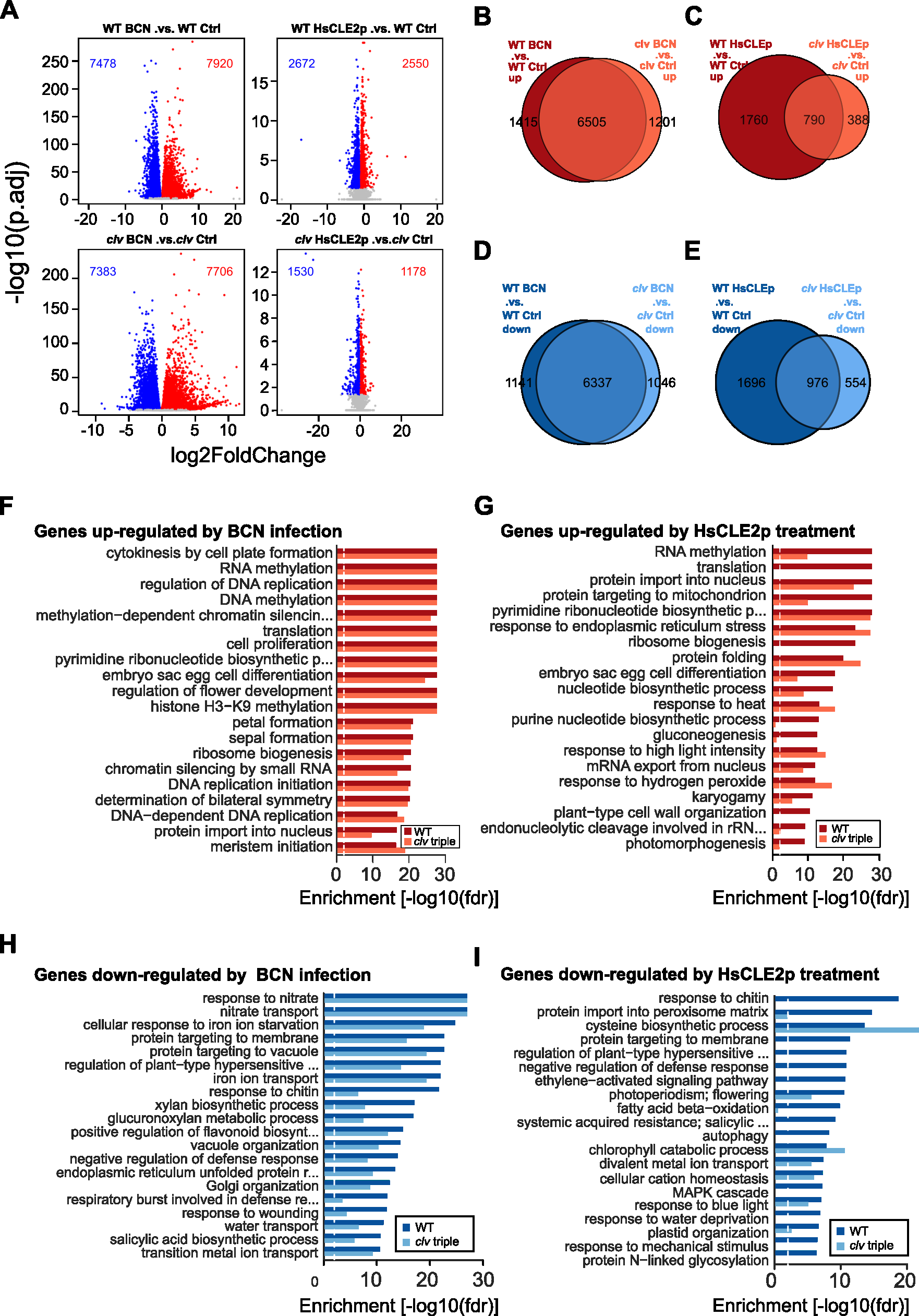

Supplement: S3 Fig — A. Volcano plot of differentially expressed genes upon BCN infection and HsCLE2p treatment in wild-type and the clv triple mutant. B—C. Venn diagram showing overlap of up-regulated genes between wild-type and clv triple mutant upon BCN infection (B) or HsCLEp treatment (C). D—E. Venn diagram showing overlap of down-regulated genes between wild-type and the clv triple mutant upon BCN infection (D) or HsCLE2p treatment (E). F, H. BCN up-regulated (F) and down-regulated (H) genes in wild-type and clv triple mutant were enriched in similar GO terms of biological process. G, I. HsCLE2p up-regulated (G) and down-regulated (I) genes in wild-type and clv triple mutant were enriched in different GO terms for biological process. (TIF) [file ppat.1012610.s003.tif]

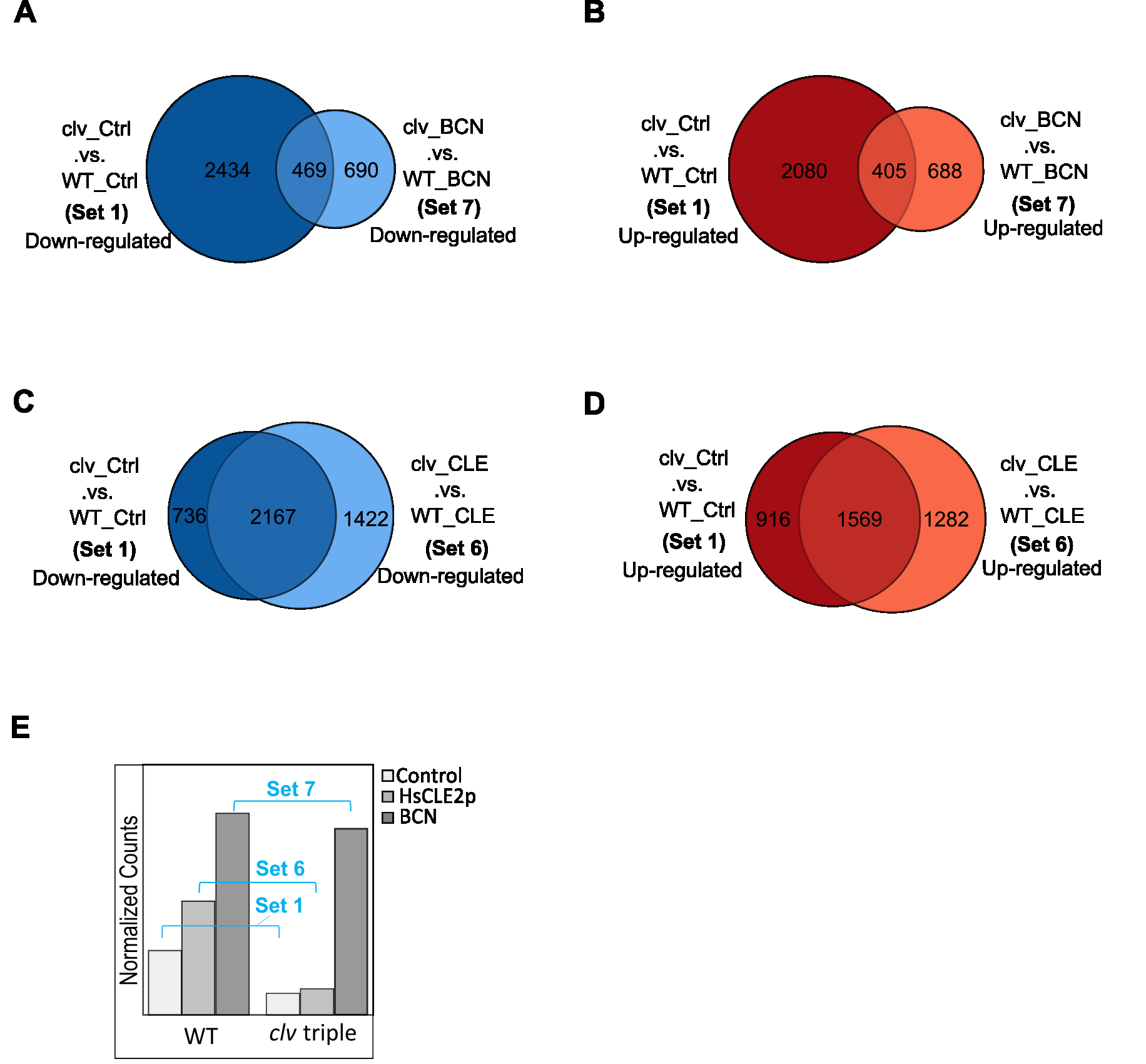

Supplement: S4 Fig — A–B. Venn diagram showing BCN infection reduced numbers of DEGs between the clv triple mutant and wild-type roots compare to control samples. C–D. HsCLE2p treatment increased numbers of DEGs between the clv triple mutant and wild-type roots compared to control samples. E. A diagram to visualize comparisons shown in panel A–D. (TIF) [file ppat.1012610.s004.tif]

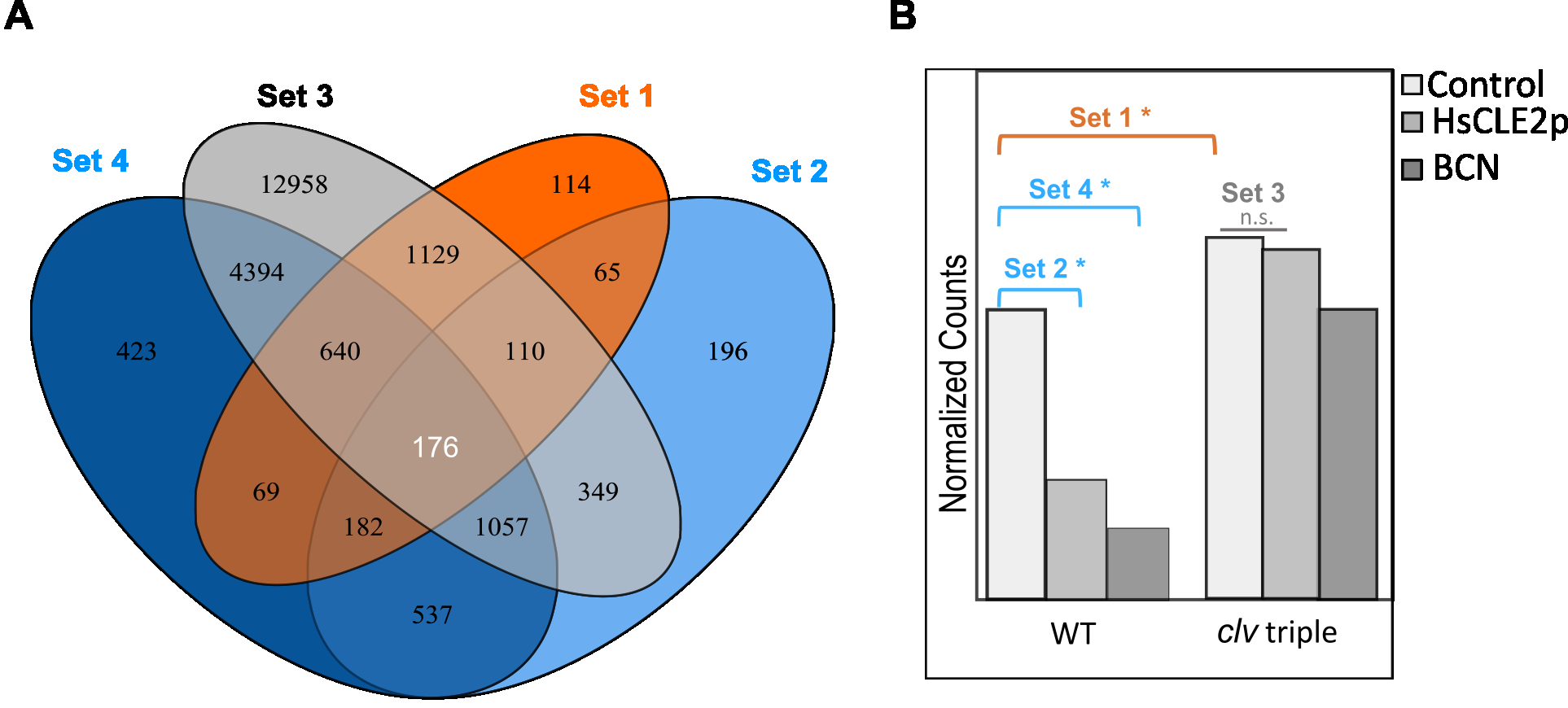

Supplement: S5 Fig — A. Venn diagram showing number of genes that are 1), Up-regulated in the clv triple mutant compared to wild-type when uninfected. 2), Down-regulated by HsCLE2p treatment in the wild-type but not in the clv triple mutant (3). 4), Down-regulated by BCN infection in the wild-type. B. A diagram showing criteria used in (A). Red font color represents up-regulated sets; Blue font color represents down-regulated sets. Gray font color represents non-significant sets. n.s., not significant; *, p < 0.05. (TIF) [file ppat.1012610.s005.tif]

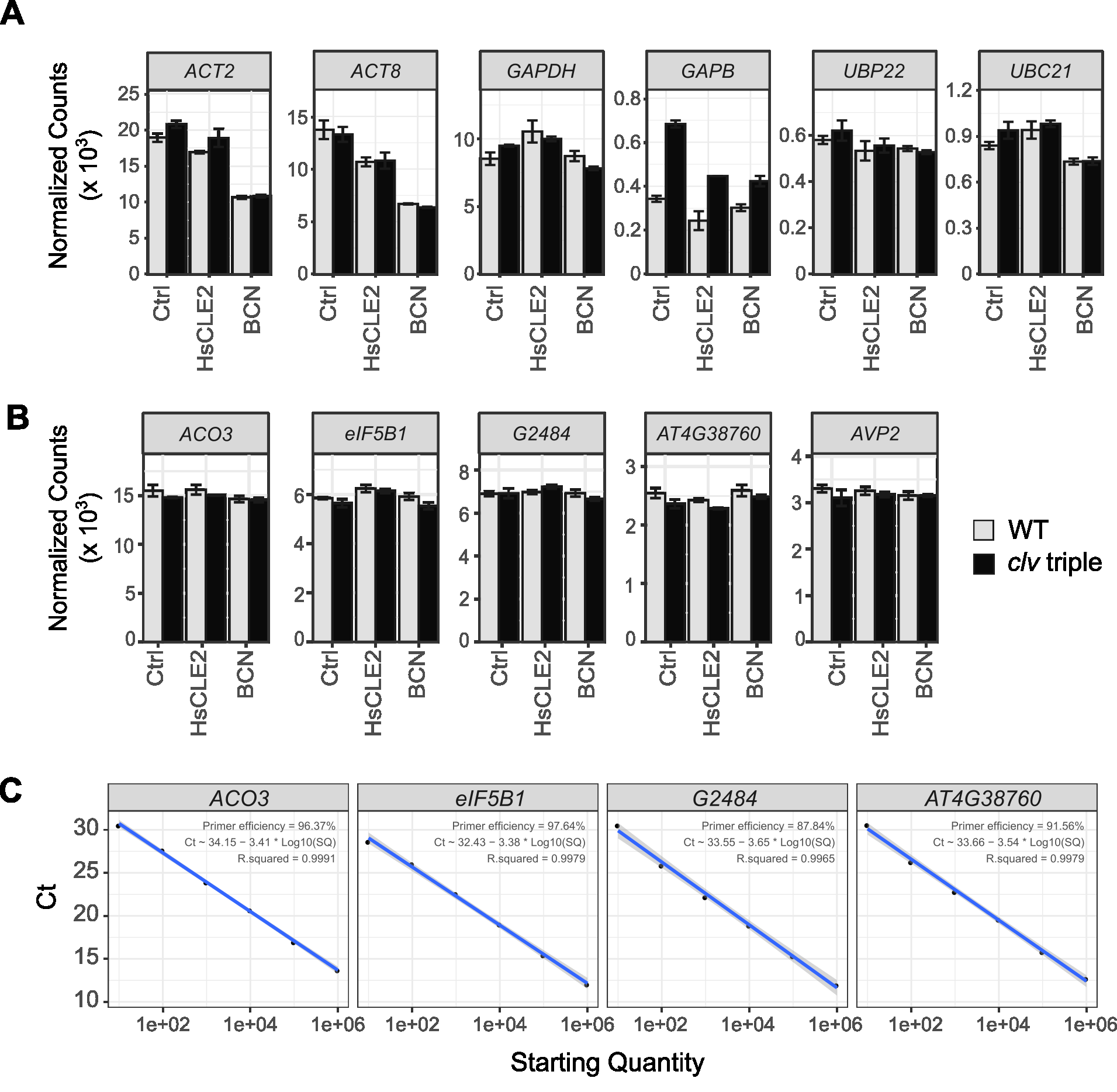

Supplement: S6 Fig — A. Expression of published reference genes, ACT2 [133], ACT8 [134], GAPDH [135], UBP22 [136, 137], UBC21 [25], and GAPB [138], in the RNAseq dataset. B. Selection of a set of new candidate genes that are consistently expressed across all samples. C. qPCR primer efficiency of selected candidate genes. The ACO3 gene is selected as the new reference gene for qPCR test. (TIF) [file ppat.1012610.s006.tif]

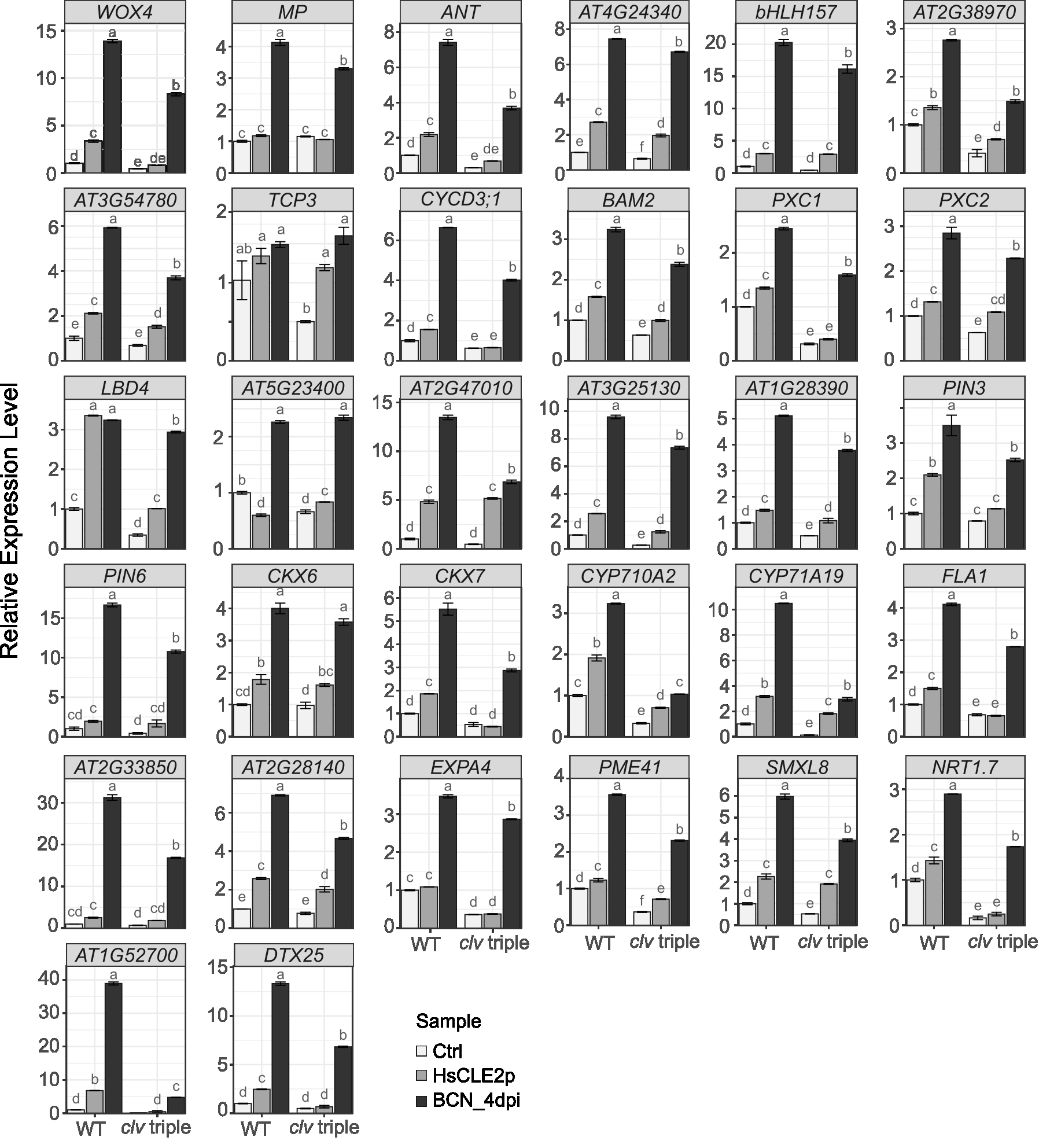

Supplement: S7 Fig — qPCR verification of a subset of tier 1 genes in S4 Dataset. Expression was verified for the rep1 and rep2 of RNAseq samples, with similar results. Only results from rep1 were shown here. Two technical replications were used for each sample. Letters above each bar graph represent statistical group of Tukey’s HSD test following ANOVA analysis. (TIF) [file ppat.1012610.s007.tif]

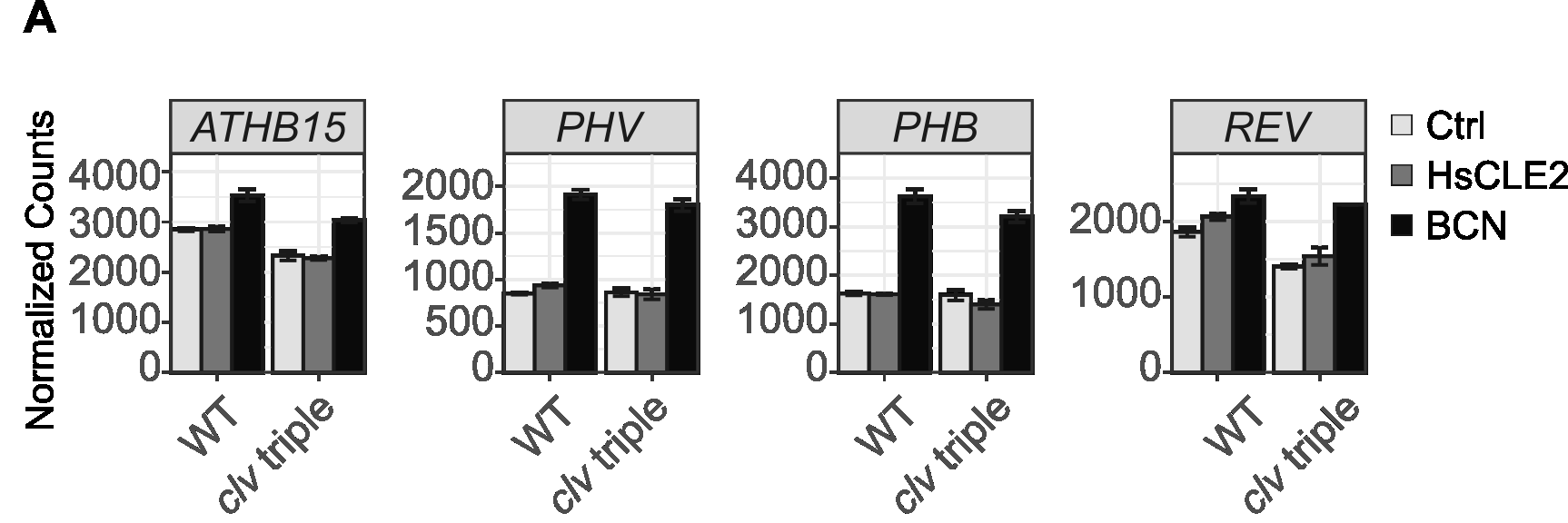

Supplement: S8 Fig — A. Expression of HD-ZIP III transcription factors in the RNAseq dataset. (TIF) [file ppat.1012610.s008.tif]

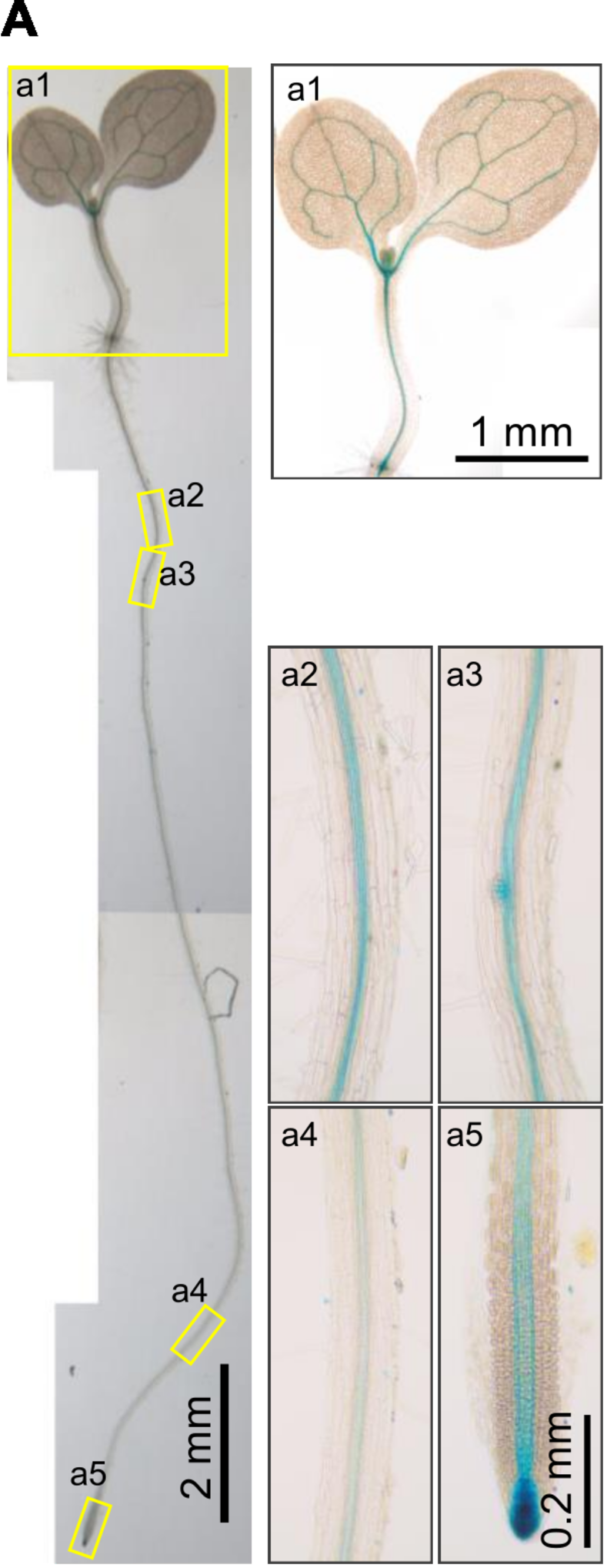

Supplement: S9 Fig — ProATHB8::GUS expression in 5-day old uninfected wild-type (Ws-2). Areas of yellow boxes were shown in the right panel at higher magnification with corresponding codes. The expression of ATHB8 gene along the root vasculature is not even. (TIF) [file ppat.1012610.s009.tif]

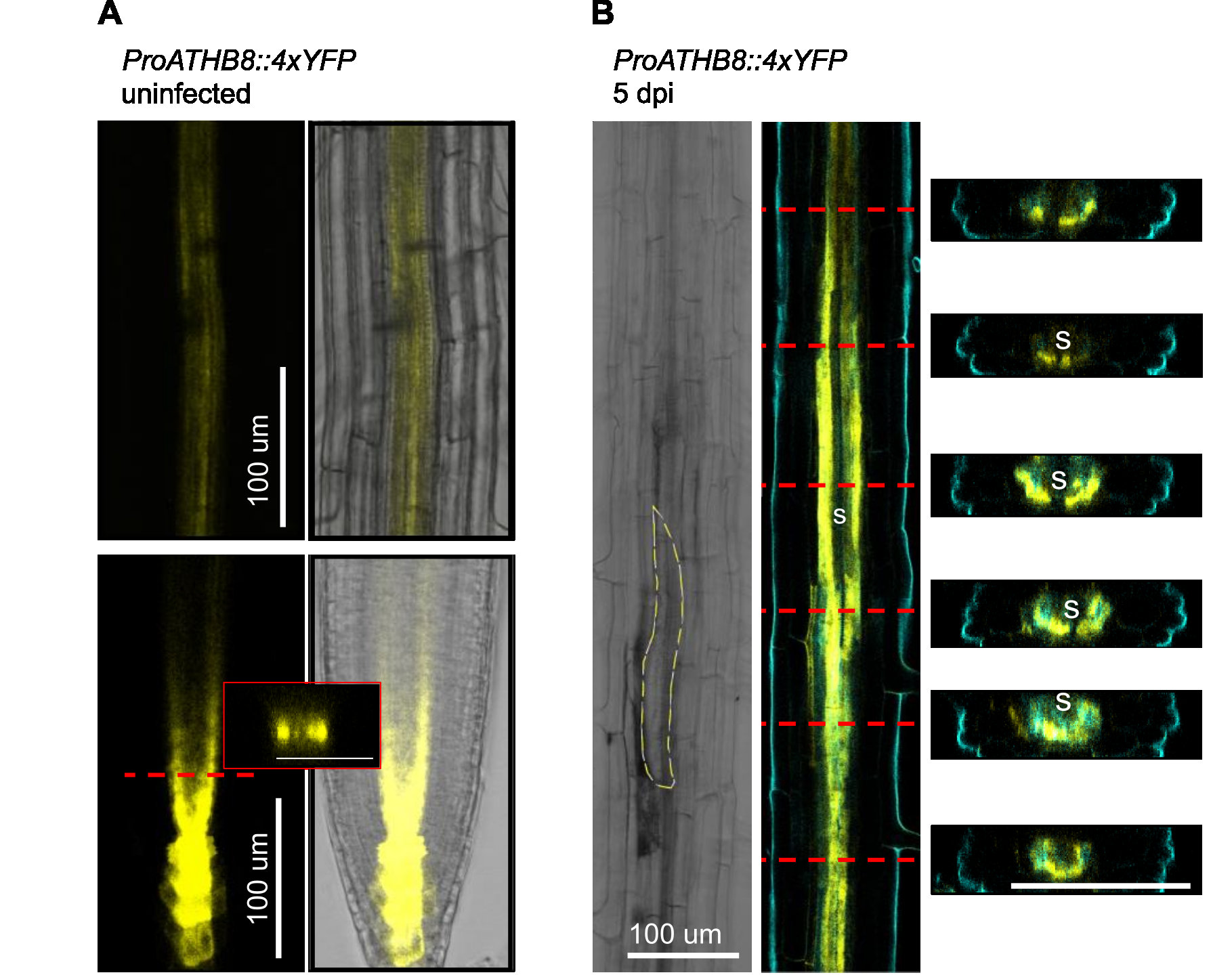

Supplement: S10 Fig — A. Expression of ProATHB8::4xYFP in 7-day-old uninfected root. A very weak YFP signal was seen in the mature vasculature (top panels) while strong YFP signal was detected in the root apical meristem (bottom panels). Insert, optical cross section of root tip showing ProATHB8::4xYFP was expressed in the xylem axis cells. B. Expression of ProATHB8::4xYFP at infection site at 5 dpi. Optical sections show concentration of YFP signal in the periphery of the syncytium. The root segment was fixed and cleared according to Kurihara et al., 2015 [132]. The yellow dashed line indicates the position of the nematode. Red dashed lines indicate the location of optical cross section. Cyan, Calcofluor White. S, Syncytium. bar = 100 μm. (TIF) [file ppat.1012610.s010.tif]

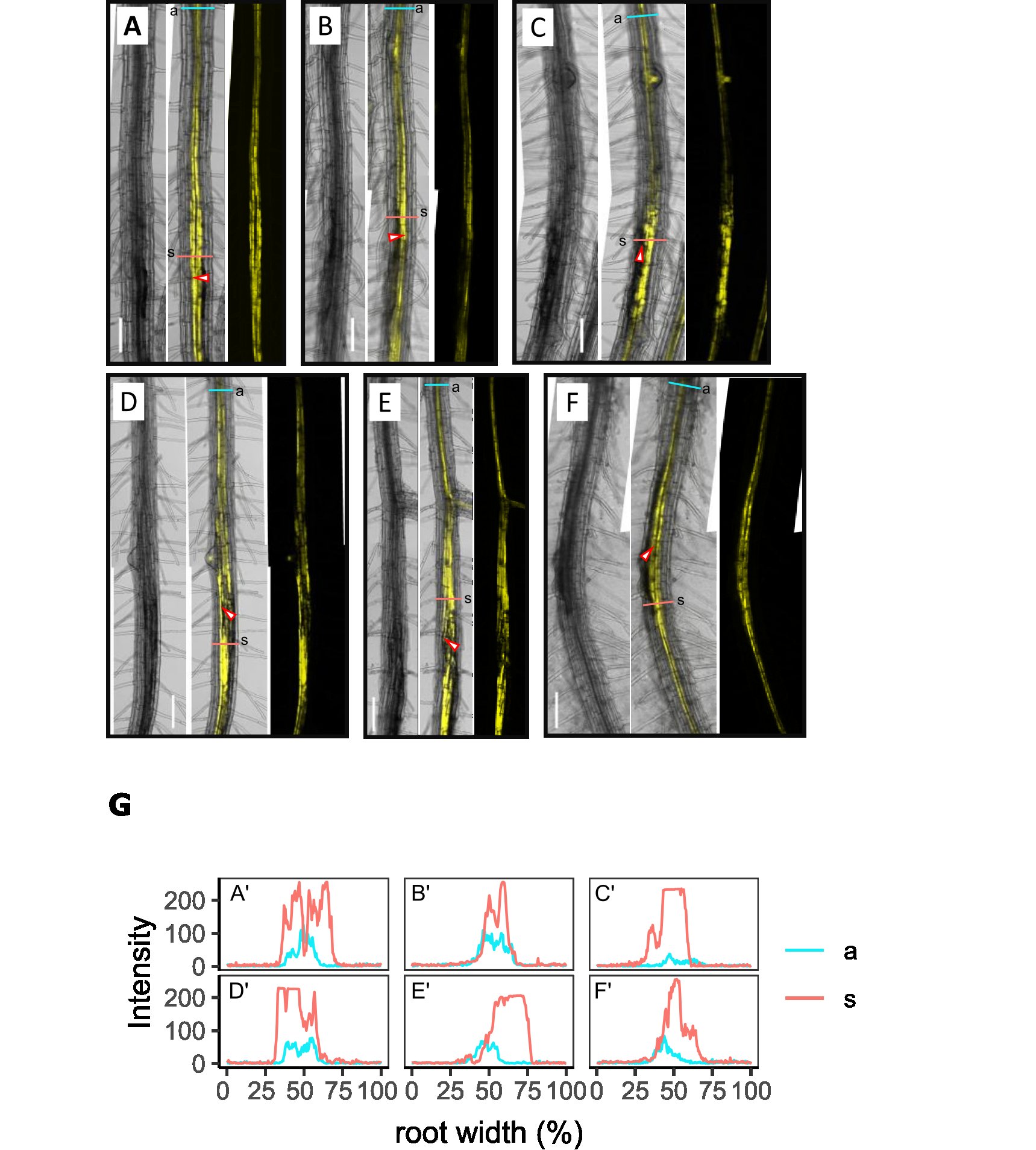

Supplement: S11 Fig — A-F, Increasing of ProATHB8::4xYFP signal in six independent BCN infection sites. G. Quantification of YFP signal intensity at syncytia (s, red line) or adjacent sites (a, cyan line) in image A-F (labeled A’-F’ respectively). Red arrowhead, position of nematode head. bar = 200 μm. (TIF) [file ppat.1012610.s011.tif]

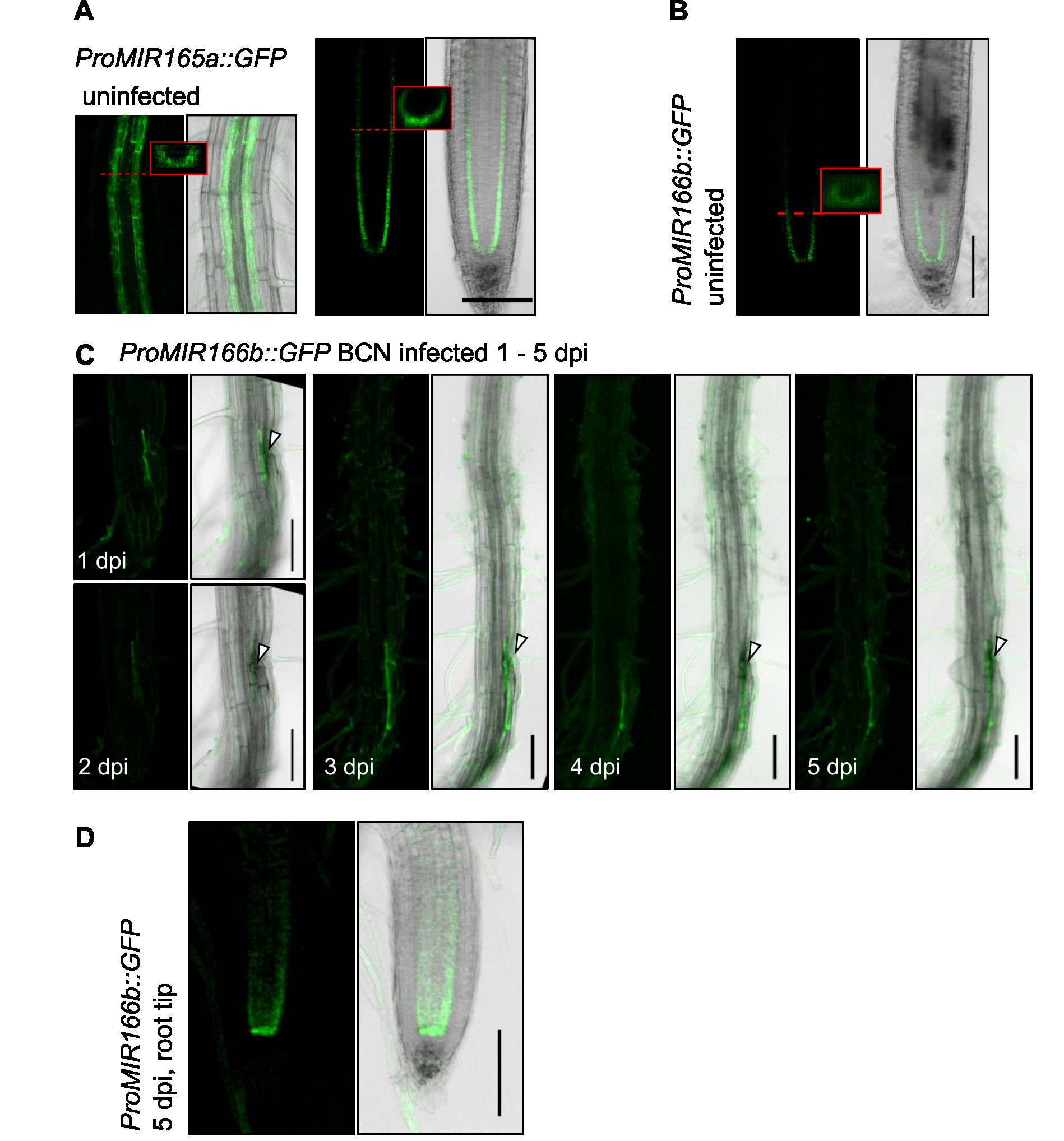

Supplement: S12 Fig — A. Expression of ProMIR165a::GFP in uninfected Arabidopsis root. GFP signal was detected in endodermis cells of both mature root (left panels) and the root tip (right panels). Optical cross section (red box) showed the expression of GFP in endodermis cells. B. Expression of ProMIR166b::GFP in endodermal cells at the root tip in uninfected root. GFP signal was not detected in mature root. C. ProMIR166b::GFP expression is not detected in BCN infection site. The same nematode was monitored from 1 to 5 days post inoculation (dpi). Green Signal near infection site is autofluorescence. D. the same seedling shown in (C) is GFP positive at the root tip (imaged at 5 dpi). Red dashed line, position of cross section. White arrowhead, position of nematode head. bar = 100 μm. (TIF) [file ppat.1012610.s012.tif]

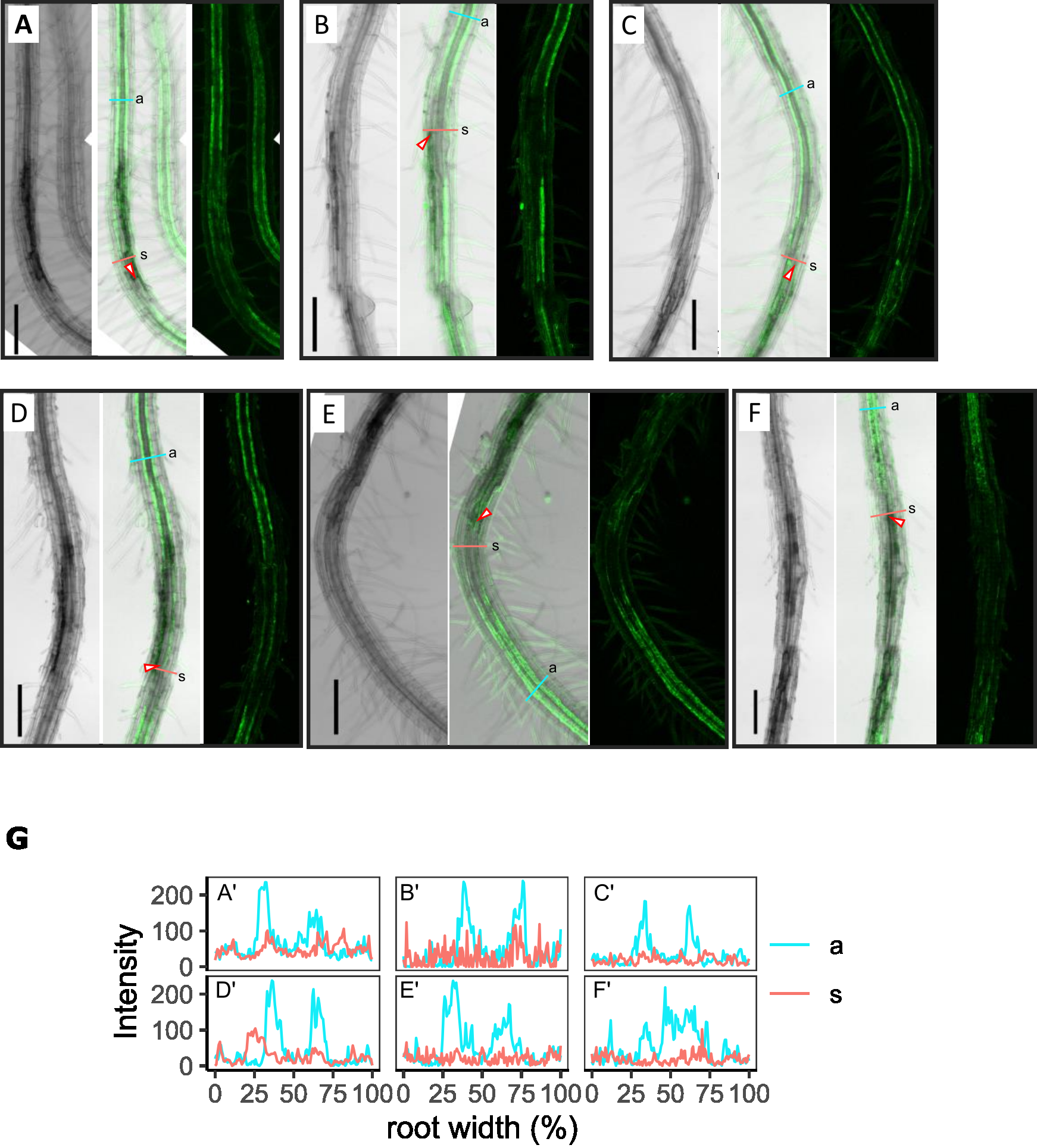

Supplement: S13 Fig — A-F, decreasing of ProMIR165a::GFP signal in six independent BCN infection sites. G. Quantification of GFP signal intensity at syncytia (s, red line) or adjacent sites (a, cyan line) in image A-F (labeled A’-F’ respectively). Red arrowhead, position of nematode head. bar = 200 μm. (TIF) [file ppat.1012610.s013.tif]

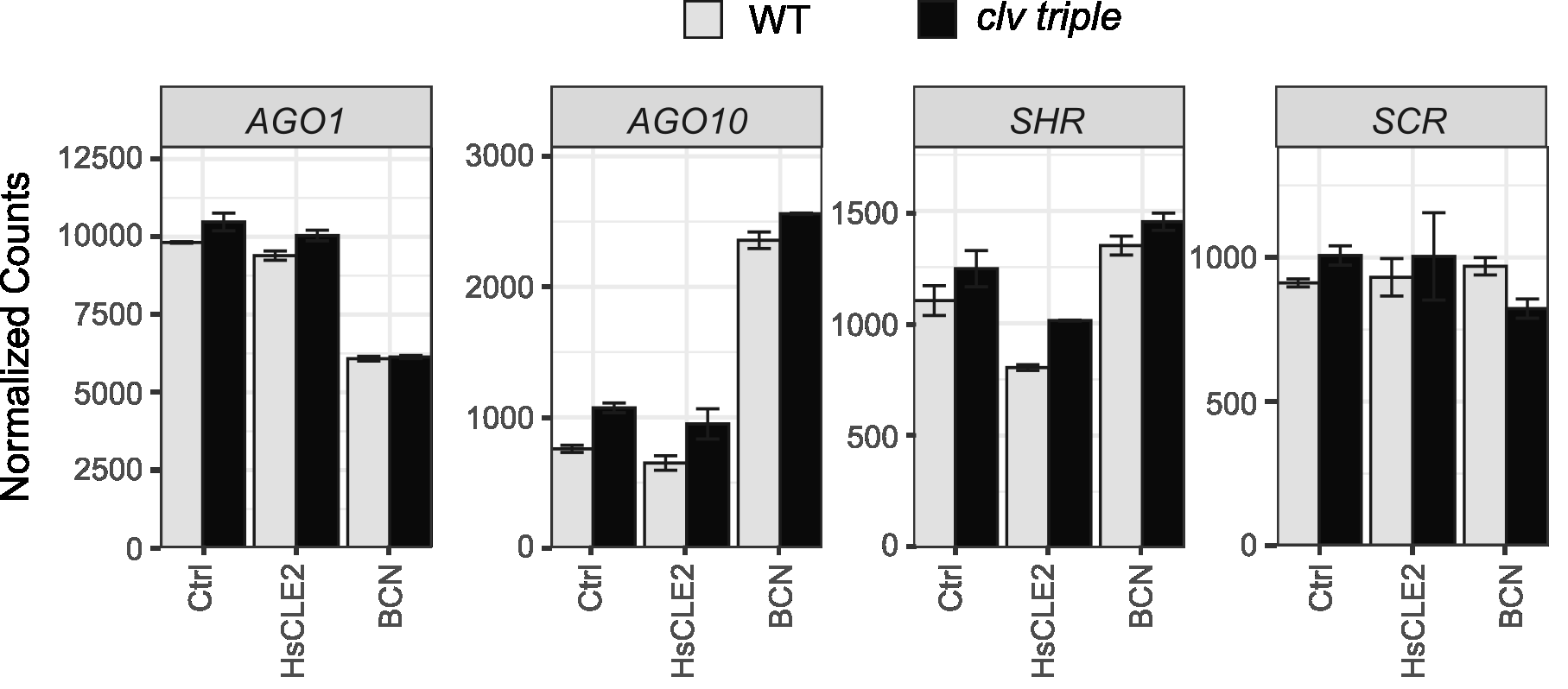

Supplement: S14 Fig — AGO1, which recruits MIR165/166 for target mRNA cleavage, is down-regulated. AGO10, which specifically sequesters MIR165/166 for degradation, is up-regulated. SHR is a stele expressed transcription regulator which moves to endodermis cells and interacts with SCR to activate MIR165/6 expression. (TIF) [file ppat.1012610.s014.tif]

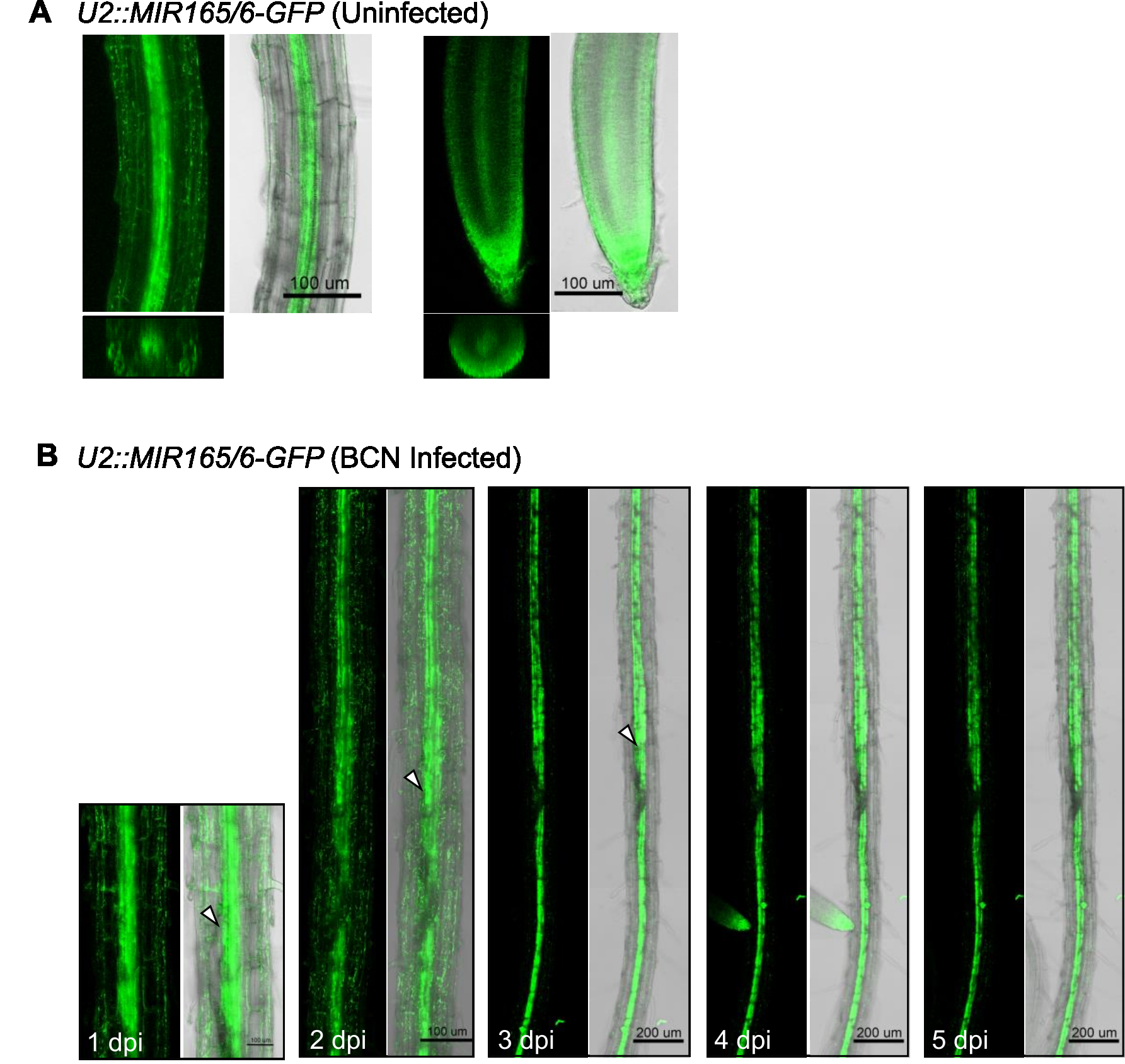

Supplement: S15 Fig — A. Expression of MIR165/6 sensor in uninfected roots. Bar = 100 μm. B. Expression of MIR165/6 sensor in the BCN infection site. The same infection site was monitored for five days. White arrowhead, position of nematode head. Bar = 100 μm for 1 and 2 dpi images, 200 μm for 3–5 dpi images. (TIF) [file ppat.1012610.s015.tif]

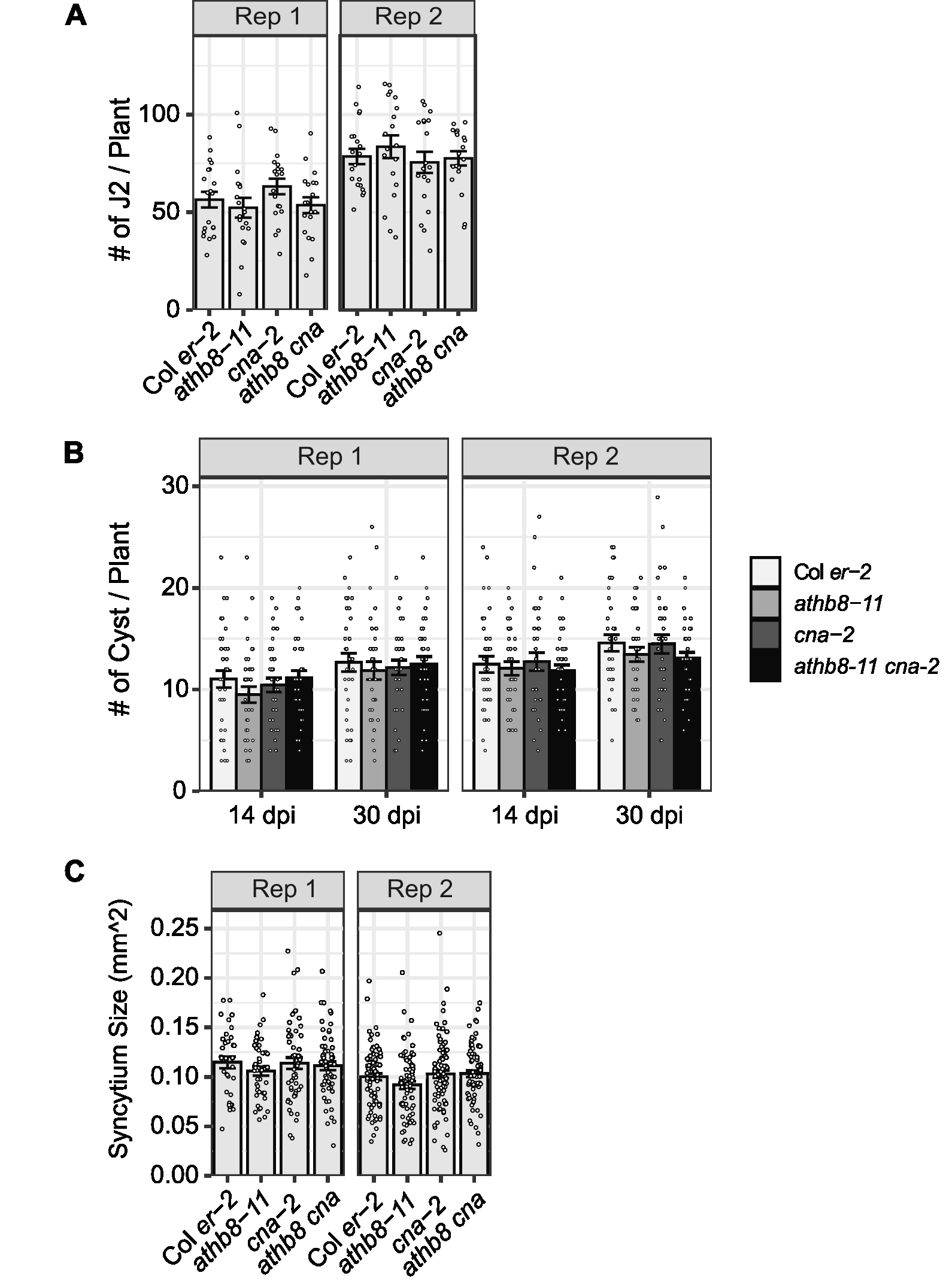

Supplement: S16 Fig — Loss of ATHB8 and/or its closest homologue ATHB15/CNA does not affect BCN penetration (A), female development (B), or syncytium size (C). Data from Rep 1 is shown in Fig 4D–4F. Bar graphs represent mean ± SE. Dots represent each individual measurement. Statistical tests were performed with Wald test following generalized linear mixed-effect model (for penetration and infection data) or linear mixed-effect model (for syncytium size). (TIF) [file ppat.1012610.s016.tif]

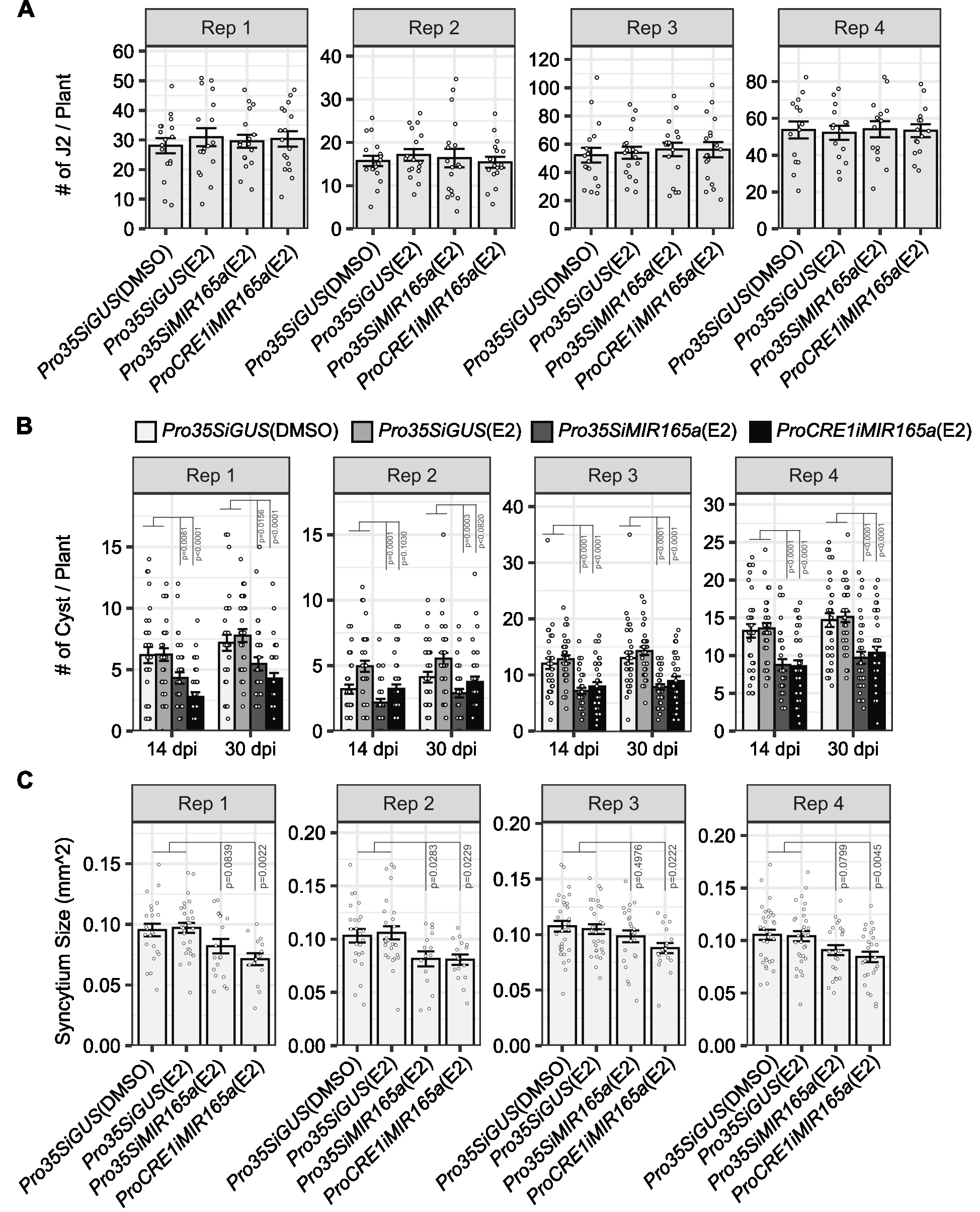

Supplement: S17 Fig — Inducible expression of MIR165a suppresses BCN female development (B) and syncytium size (C), but not BCN penetration (A) in Arabidopsis. Data from Rep 3 of (A-B) is shown in Fig 5C and 5D. Combined data from (C) is shown in Fig 5. Bar graphs represent mean ± SE. Dots represent each individual measurement. Statistical tests were performed with Wald test following generalized linear mixed-effect model (for penetration and infection data) or linear mixed-effect model (for syncytium size). Only statistically significant results were labeled. (TIF) [file ppat.1012610.s017.tif]

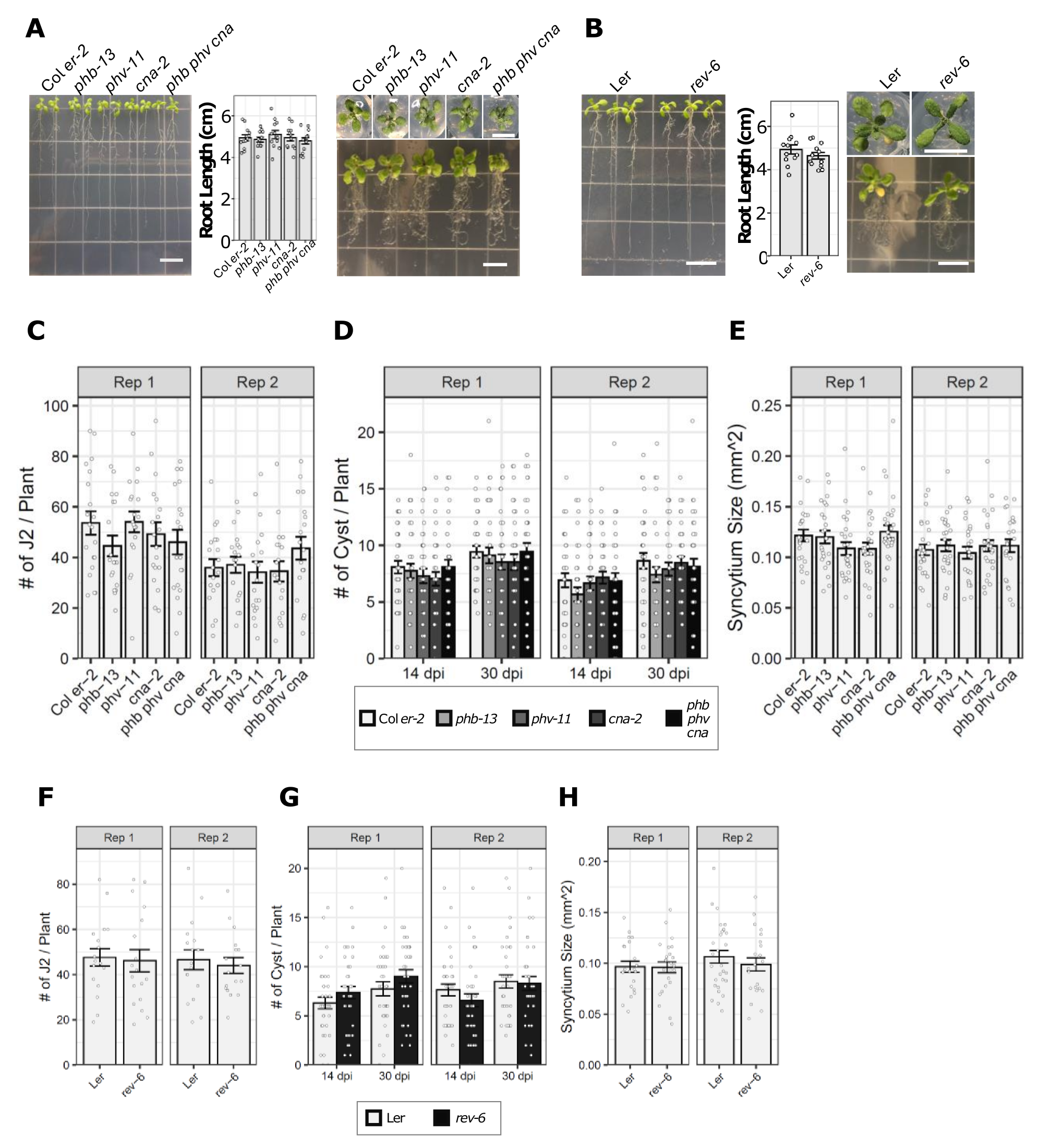

Supplement: S18 Fig — A. phb-13, phv-11 single and phb-13 phv-11 cna-2 triple mutants didn’t show significant root growth defect in both vertical plates (left and middle panel) and 12-well plate (right panel). B. rev-6 mutant did not show significant root growth defect in both vertical plates (left and middle panel) and 12-well plate (right panel). C-E. phb-13, phv-11, and cna-2 mutants as well as their triple mutant didn’t show significant different in BCN penetration (C), female development (D), and syncytium size (E) compared to that of wild-type (Col er-2). F-H. rev-6 mutant didn’t show significant different in BCN penetration (F), female development (G), and syncytium size (H) compared to that of wild-type (Ler) Statistical tests were performed with Wald test following generalized linear mixed-effect model (for penetration and infection data) or linear mixed-effect model (for syncytium size). bar = 1 cm in A and B. (TIF) [file ppat.1012610.s018.tif]

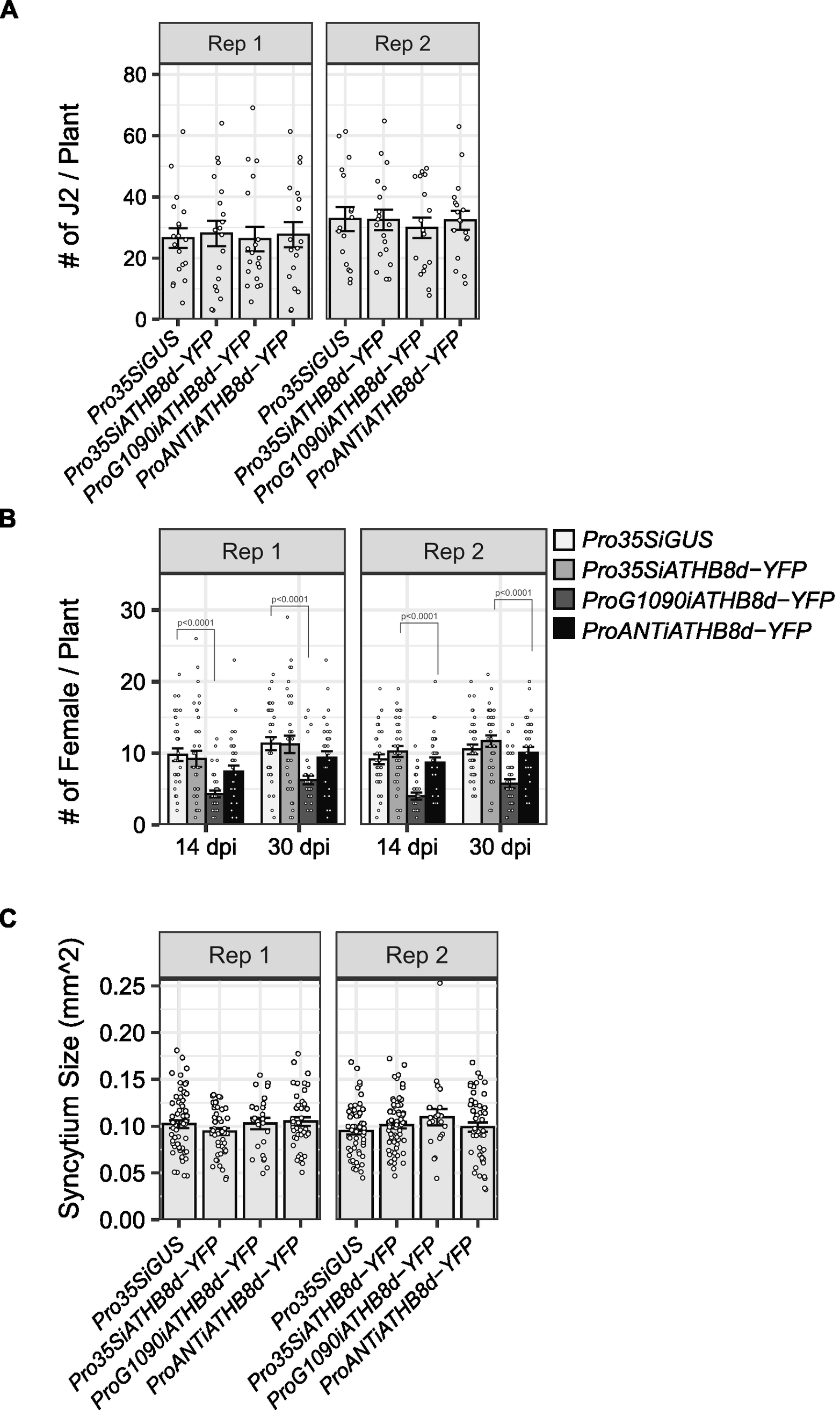

Supplement: S19 Fig — Effect of inducible overexpression of ATHB8d-YFP on BCN penetration (A), female development (B), and syncytium size (C) in Arabidopsis. Data from Rep 1 is shown in Fig 6 (C-E). Bar graphs represent mean ± SE. Dots represent each individual measurement. Statistical tests were performed with Wald test following generalized linear mixed-effect model (for penetration and infection data) or linear mixed-effect model (for syncytium size). Only statistically significant results were labeled. (TIF) [file ppat.1012610.s019.tif]

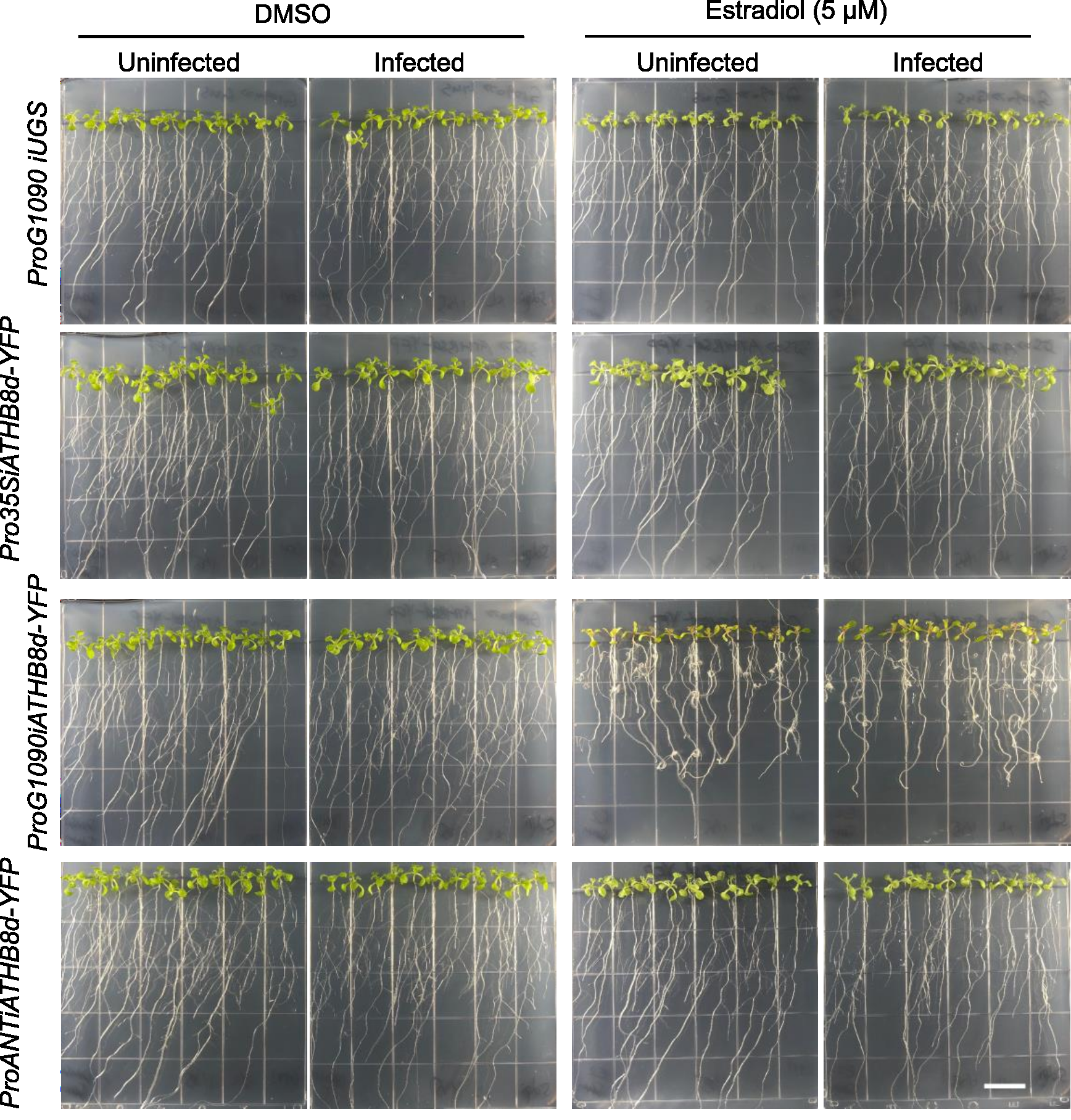

Supplement: S20 Fig — Seedlings were grown on vertical plates with Knop’s media for 5 days, then were moved to Knop’s with DMSO or 5 μM Estradiol. Two days after estradiol induction, each root was inoculated with about 15 J2s of BCN. Plates were imaged five days after inoculation. The ProG1090iATHB8d-YFP line showed distorted root development after estradiol induction with or without BCN infection. bar = 1cm. (TIF) [file ppat.1012610.s020.tif]

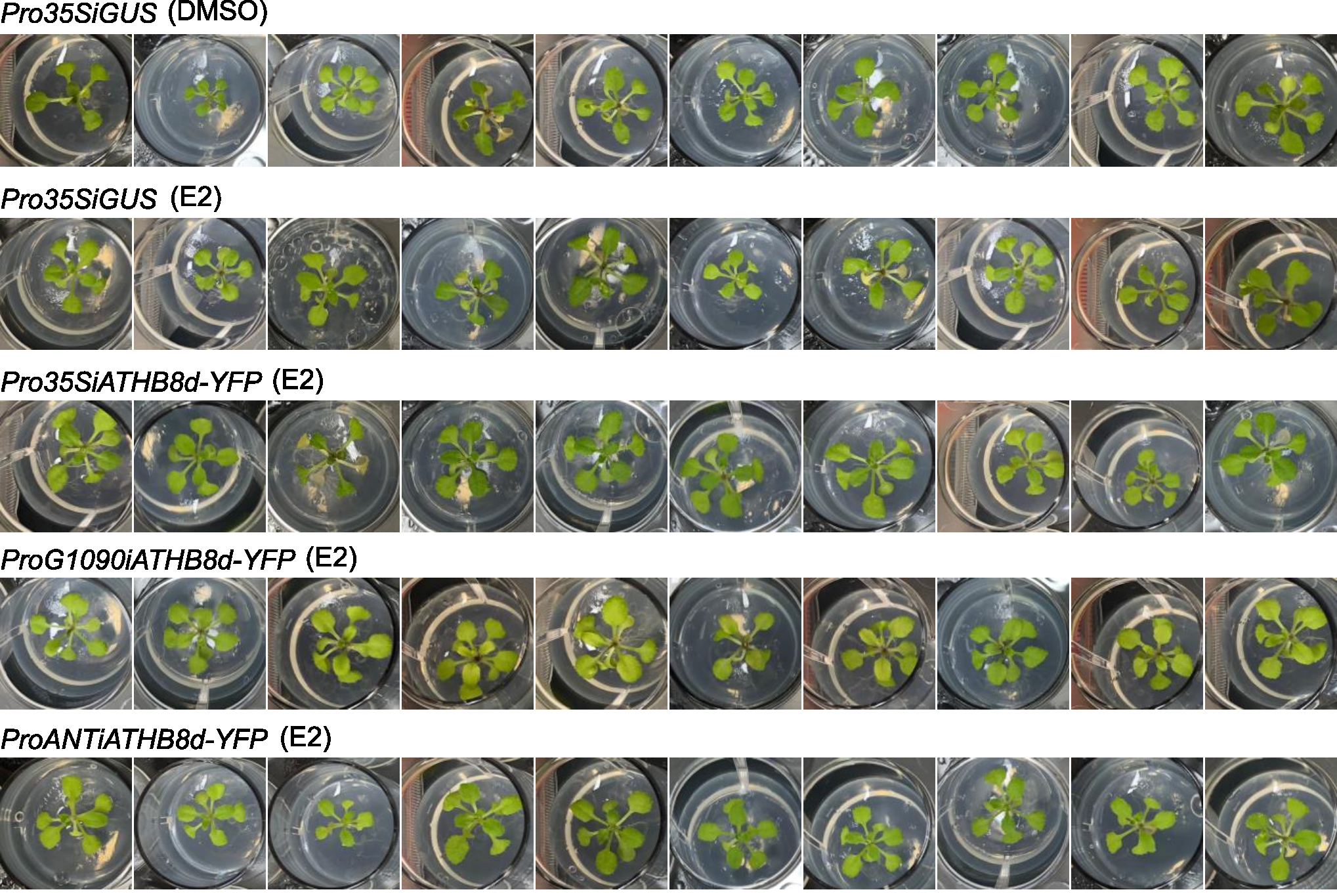

Supplement: S21 Fig — 18 seedlings for each genotype/treatment combination were grown in 12-well plates with Knop’s media. 5 μM estradiol or an equivalent amount of DMSO were added to each well at 12 days post germination. Two days after estradiol induction, each well was inoculated with about 200 J2s of BCN. Plates were imaged 5 days after inoculation. Images of 10 seedlings from each genotype/treatment combination were shown. E2, estradiol. (TIF) [file ppat.1012610.s021.tif]

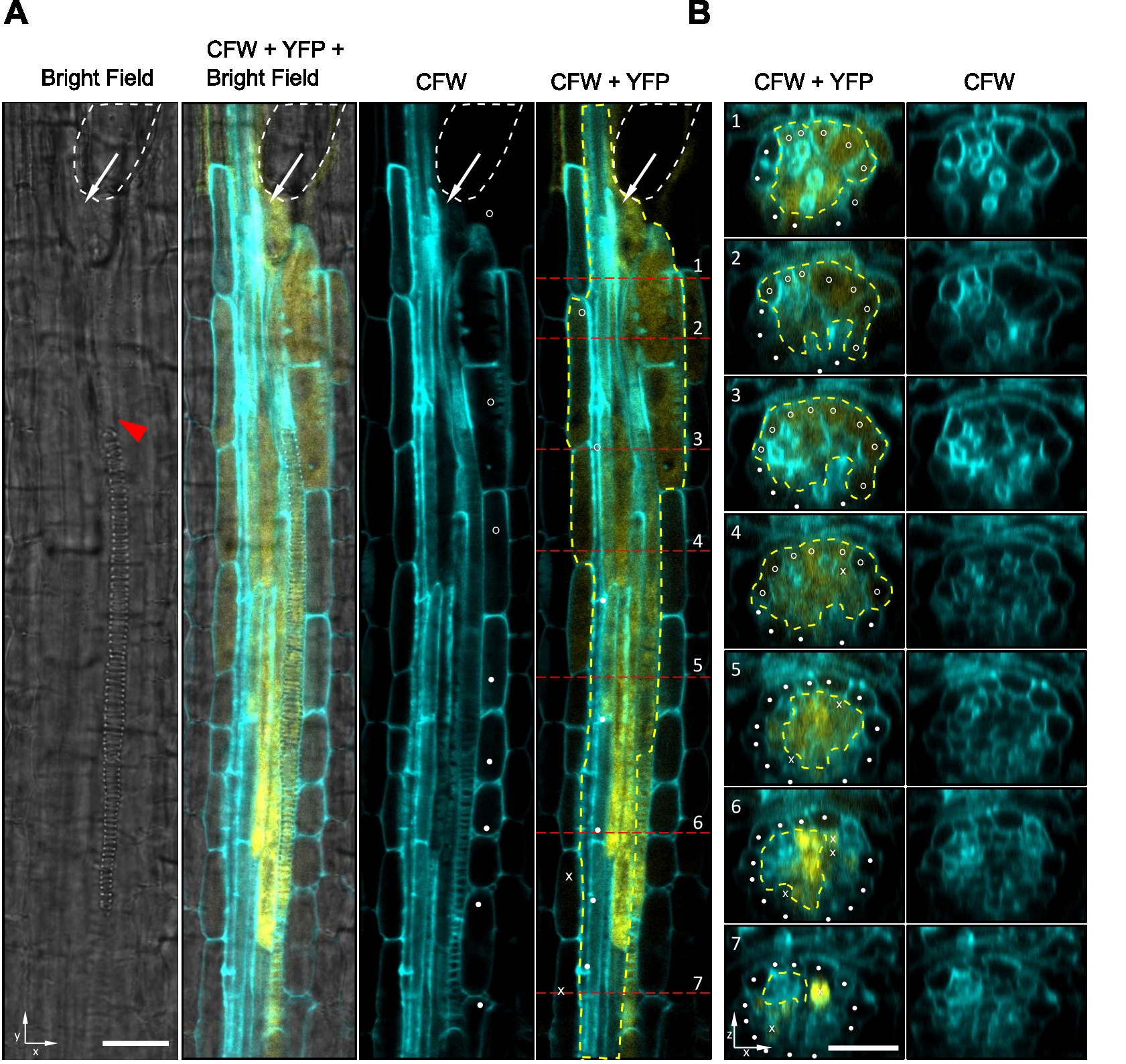

Supplement: S22 Fig — A. A confocal optical section of developing syncytium with DR5::4xYFP expression. B. Optical cross sections of positions shown in (A). White dashed line, outline of nematode head. White arrow, position of the stylet. Red dashed line, positions of cross section shown in (B). Yellow dashed line, outline of the syncytium. Red arrowhead, disrupted xylem differentiation. x, xylem cells. White dot, pericycle cells. White circle, pericycle cells incorporating into the syncytium. CFW, calcofluor white staining of cell walls. YFP, yellow fluorescent signal. bar = 20 μm. (TIF) [file ppat.1012610.s022.tif]

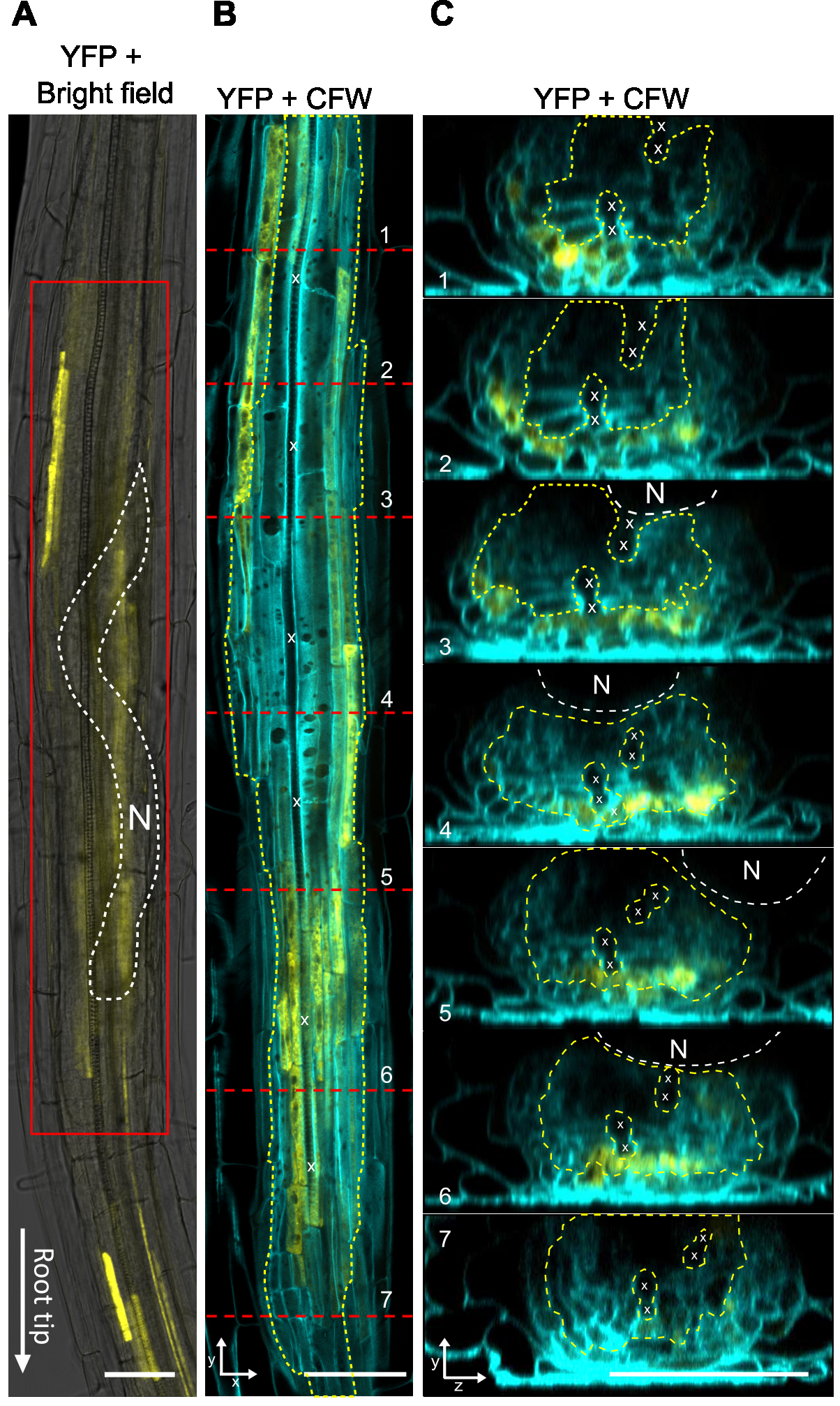

Supplement: S23 Fig — White dashed line, outline of nematode. Red box, zoomed in portion shown in (B). Red dashed lines in (B), position of optical cross sections shown in (C). Yellow dashed line, outline of the syncytium. x, xylem cells. CFW, calcofluor white staining of cell walls. YFP, yellow fluorescent signal. bar = 50 μm. (TIF) [file ppat.1012610.s023.tif]

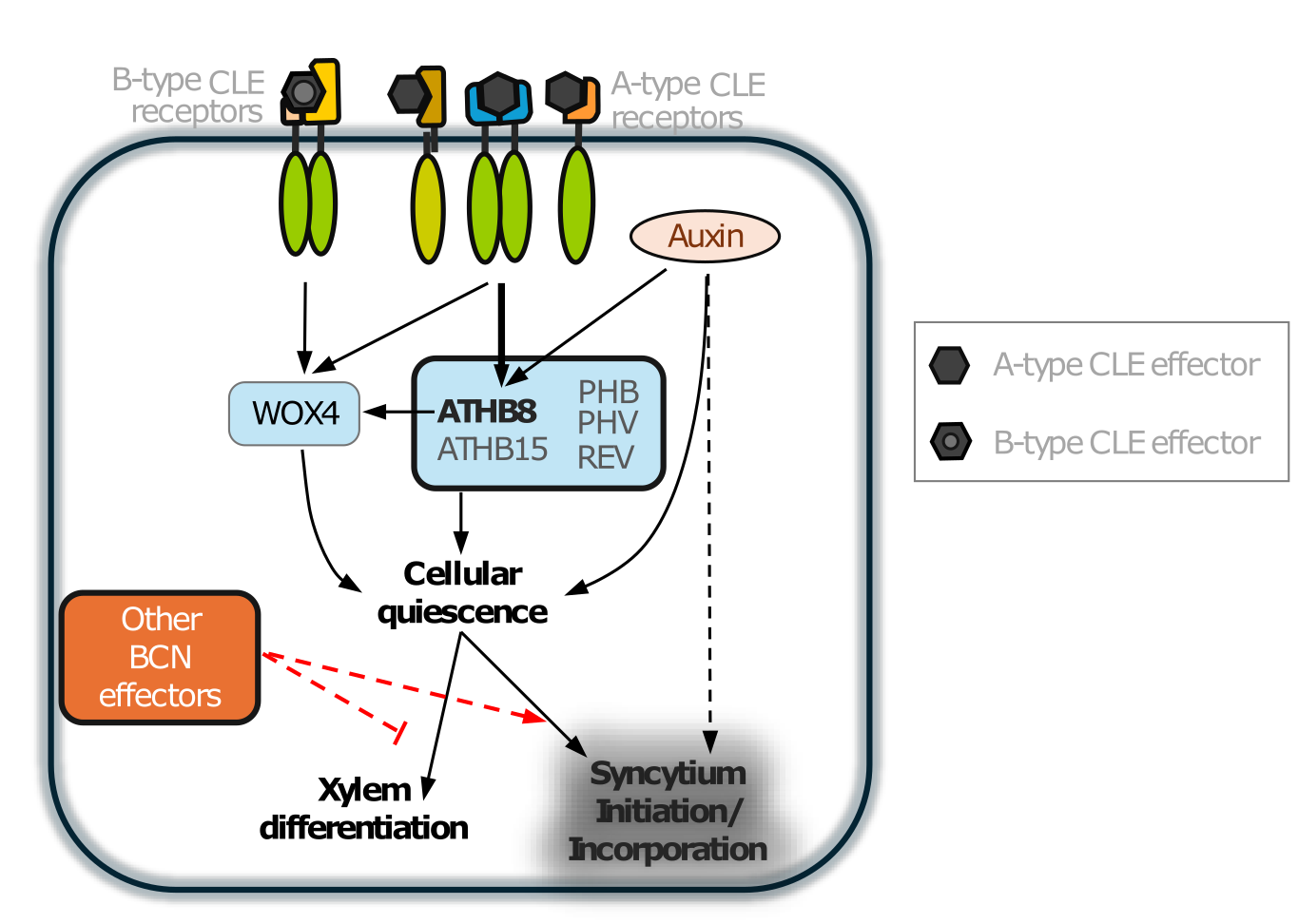

Supplement: S24 Fig — High HD-ZIP III gene expression promotes plant cells into quiescent status with partial xylem identity known as a stem cell organizer, indicating that the nematode may use a stem cell organizer as an intermedia status for syncytium initiation/incorporation. Other BCN effectors may be needed to suppress xylem differentiation and/or promote the transit of a stem cell organizer into a syncytial cell. (TIF) [file ppat.1012610.s024.tif]
