# Supplementary material for: A major role of class III HD-ZIPs in promoting sugar beet cyst nematode parasitism in Arabidopsis
Source: PLoS Pathog. 2024 Nov 7;20(11):e1012610. doi: 10.1371/journal.ppat.1012610 (PMC11542791; doi:10.1371/journal.ppat.1012610)
Supplement: Attachment — Submitted filename: Liu, Mitchum_Reponse to reviewers.docx [file ppat.1012610.s032.docx]

**Response to Reviewers**

**Reviewer #1:**

**Part I - Summary**

The manuscript by Liu et al. identifies and characterizes ATHB8, which encodes a HD-ZIP III family transcription factor, as a downstream component of the CLE signaling pathway in syncytium formation. ATHB8 is expressed in the syncytium during the early stages of infection and then transitions to neighboring cells of the syncytium as it expands, showing an expression pattern coincident with auxin response at the infection site. Knocking down HD-ZIP III by over-expressing MIR165a reduced the female development of the cyst nematode. Overall, the manuscript is well-written, the data seems rigorous, and it provides an important advance in understanding cyst nematode-induced syncytium formation.

**Part II – Major Issues: Key Experiments Required for Acceptance**

(1) The authors used a time point of 4 days after inoculation for their transcriptome study. It would be helpful if the authors could explain why they chose this specific time point.

>Our focus for this study was on the early stages of syncytium formation. Following inoculation with infective second-stage juveniles (J2), it can take between 12-24 hours for the juveniles to locate, penetrate, and then migrate through roots until they reach a cell near the vasculature where they will stop and initiate the formation of a syncytium (feeding site). Four days post-inoculation represents an early timepoint. At four days post-inoculation of Arabidopsis roots, J2 will be at different stages of the infection process, with syncytia ranging anywhere from 24-72 hrs old. This was the earliest timepoint infection sites could be clearly seen using a stereoscope for precise tissue excision to enrich for syncytia.

(2) Lines 207-214: The authors suggest that the transcriptomic similarity between the clv triple mutant and wild-type samples in response to BCN infection indicates that BCN-infected samples might not be optimal for filtering out downstream genes of the CLE signaling pathway. Instead, they propose that HsCLE2p treatment would be a more effective approach. While I understand the rationale, I would argue that a reduced number of DEGs between the clv triple mutant and wild-type is advantageous as it highlights fewer but biologically relevant differences. In contrast, exogenous peptide treatment, where all root tissues are treated with a peptide at a potentially non-physiological concentration, might not provide the same relevance. Perhaps the authors could clarify this point further.

>We agree that the reduced number of differentially expressed genes (DEGs) between the clv triple mutant and wild-type samples in response to BCN infection can indeed highlight biologically relevant differences. This attenuation of gene expression suggests that BCN is capable of triggering a robust compensatory mechanism in the *clv* triple mutant to allow the establishment of syncytium even in the absence of key CLE receptors.

However, this compensatory mechanism is precisely what we were concerned about in filtering for CLE downstream genes. It should be noted that excised infection sites for RNA-seq already have some successfully established syncytial cells, which means the disrupted CLE signaling pathway was circumvented, likely through alternative or parallel pathways. This may obscure the identification of genes directly regulated by CLE signaling.

On the contrary, HsCLE2p treatment provides a controlled approach to specifically activate the CLE signaling pathway. While we acknowledge that exogenous peptide treatment might not fully replicate physiological conditions, it allows us to isolate and study the direct effects of CLE signaling on gene expression without the confounding influence of other nematode effectors. In addition, we also optimized peptide concentrations and treatment times using the known CLE downstream gene *WOX4* to best mitigate potential non-physiological effects.

Overall, we believe the HsCLE2p treatment is a more effective approach to isolate genes regulated by the CLE signaling pathway.

(3) Line 221: Including this methodology in Figure 2 would be helpful to readers.

>The methodology is shown in Figure 1B (previously Figure 2A). Filtering criteria is described in the corresponding figure legend.

(4) Lines 300-309: Upon BCN infection, the expression of ProMIR165a::GFP signal is gradually reduced at the infection site. I observed expression only in endodermal cells around the feeding site, which seems to disappear after 5 days. The authors should clarify the significance of this expression pattern more thoroughly. In general, I found the expression pattern of MIR165a::GFP a bit puzzling.

>MIR165a itself is expressed in the endodermal cells and moves into the stele to suppress HD-ZIP III TFs (Carlsbecker et al., 2010. Nature 465:316–321). Thus, our observation is consistent with the previous report. MIR165a expression pattern is mentioned in the abstract (line 34), line 269 before describing its expression at BCN infection site. The significance of this expression pattern is discussed in the discussion section (lines 488-492).

(5) Figure 4a vs. 4b: There is a difference in the expression pattern between Figures 4a and 4b. The authors should address this difference explicitly in results section i.e. MIR165a expression in endodermis and transitioning..

>The *ATHB8* gene is expressed in the stele, and MIR165a is normally expressed in the endodermis and moves into the stele to suppress the expression of *ATHB8* and other HD-ZIP III genes. Our result is consistent with these expression patterns. The expression pattern of MIR165a is explained in the text (line 269).

(6) Figure 5: Please add to the figure legend how many times the experiment was independently replicated.

>It was repeated twice with similar results and this has been added to the figure legend.

**Part III – Minor issues**

Including a model at the end of the manuscript would help explain the findings to the audience.

>We have included a model to summarize the findings (Supplemental Figure S24).

**Reviewer #2:**

**Part I - Summary**

In this manuscript, Liu et al. analyzed the role of the CLE signaling pathway in syncytium formation and revealed the function of HD-ZIP III TFs, including ATHB8, in connecting the CLE and auxin signaling pathways to promote syncytium formation. The authors provide an interesting and well written paper. Their dataset is informative and communicated in the context. However, some results need to be improved. My comments on the manuscript are as follows.

**Part II – Major Issues: Key Experiments Required for Acceptance**

1. What is the best time point at which BCN induces the most significant DEGs? The authors need to test this by experiments, or it is not clear to the readers whether the less DEGs results from no or over induction by BCN in Figure 1. The authors need to discuss this at least.

>We would like to clarify that there are mainly two groups of DEGs discussed in this manuscript: 1). DEGs between BCN infection and uninfected samples in WT or mutant, and 2). DEGs between clv triple mutant and WT after BCN infection or HsCLE2p treatment.

>We do not show “less DEGs” in the previous Fig 1C-D and Fig S3A-B (now Fig S3B-E), but rather we showed the first type of DEGs (with/without BCN infection, or with/without HsCLE2p treatment), and that BCN infection has more overlapping DEGs in WT and mutant compared to HsCLE2p treatment. In Fig S4, we showed that there are less DEGs between BCN infected *clv* mutant and BCN infected WT (clv_BCN vs. WT_BCN) compared to uninfected mutant and WT (*clv*_Ctrl .vs. WT_Ctrl) (Figure S4A-B). We think this is mainly due to BCN’s ability to activate or even overcompensate CLE downstream genes through parallel or alternative signaling pathways (like B-type CLE signaling, auxin, and cytokinin, etc.). It is also worth noting that samples collected from the clv triple mutant have some syncytial cells, whereas failed infection sites (which are more prevalent in the mutant) cannot be collected, so this also reduces the detection of differences between WT and the mutant.

>Regarding the best time point to get most significant DEGs, it depends on which group of DEGs are of interest. In the case of the 2^nd^ group of DEGs (between *clv* mutant and WT during BCN infection), the best time would be right before syncytia degenerate in the *clv* triple mutant (if it ever forms in the *clv* triple mutant). Nevertheless, such a time point is difficult to catch and collecting such tissue is almost impossible.

2. The authors analyzed the protein localization using transgenic plants, however, whether these fused protein are expressed correctly remains unclear. They should test both the mRNA expression level and the protein level in the transgenic plants to confirm their results that the localization or accumulation are affected by the BCN. For example, the correct expression of ATHB8-YFP is very important for drawing conclusions.

>We apologize for the confusion. We would like to clarify that we did not analyze the protein localization of ATHB8-YFP, rather, we analyzed *ATHB8* promoter activity using the transcriptional fusion construct *ProATHB8::4xYFP*. It would be interesting to analyze ATHB8 protein level at the infection site, nevertheless, the transgenic lines we obtained from ABRC have no visible signal under confocal.

3. Line 363: These results are confusing. The expression of HD-ZIP III TFs was not significantly downregulated, but the BCN infection phenotype was very obvious. How to explain the phenotype vs. gene expression levels? If so, the athb8 cna double mutant should show the same phenotype with no difference in infection rate.

>In Figure 5 A-B (previously Fig 6 A-B), we showed that all HD-ZIP III’s were significantly downregulated at 2-5 days post-induction (0-3 days after BCN inoculation), which is a critical time window for the nematode to establish an infection site. While it is ideal to evaluate success of BCN infection rate at an early infection stage, there’s no reliable marker or phenotype to do so, and successfully established infection sites could also degenerate later on. As such, successful BCN infection is evaluated by BCN female development at 14 days post-inoculation. Thus, the BCN infection phenotype shown at 14 dpi was caused by down-regulation of HD-ZIP III genes at an early stage of BCN infection.

*athb8 cna* has no phenotype due to functional complementation from other HD-ZIP III family members, *PHB*, *PHV*, and *REV*.

**Part III – Minor issues**

1. Line 237 and Figure S6: Why ATHB8 is a strong candidate that was chosen by the authors? The change in ATHB8 expression was not significant compared to that of several other genes.

>We did identify several interesting candidates. The decision to pursue *ATHB8* for functional analysis was based on its expression pattern and essential function in vascular cell differentiation. Additionally, HD ZIP III’s were not previously implicated in cyst nematode infection.

2. Figure 4: The control should be added in this Figure. Additionally, for clarification, quantitative analysis of YFP/GFP signals should be performed.

>We used the region adjacent to the infection site for direct comparison, as the expression of *ProATHB8::YFP* is not even along the root, such that an adjacent root segment provided the best reference for normal expression level. An uninfected control is provided in Fig S10A.

>Quantification of both *ProATHB8::YFP* and *ProMIR165a::GFP* is now added to the 3 dpi panel in Figure 3 (previously Fig 4) and quantification of additional infection sites for both markers are provided in Fig S11 and Fig S13.

3. Figure 6B and 6D: The expression of ATHB8 and its homologs was not suppressed at 14 dpi, but the number of females in these plants also decreased. Why?

>The number of females at 14 dpi is dependent on the successful establishment of feeding cells at an early stage of BCN infection (approximated 1-5 dpi), at which point HD-ZIP III genes are suppressed in both *35S::XVE>>MIR165a* and *ProCRE1::XVE>>MIR165a*. There’s no reliable phenotype or biomarker to evaluate successful feeding cell establishment at early stages of BCN infection.

4. All Figures should be referenced in order of appearance.

>Figure orders are changed according to their order of appearance.

5. Line 187: The number was wrong. 30.98% (790 out of 2,550)?

>Thank you for pointing out this mistake, the numbers are now corrected (now line 182)

6. Line 192: The recognition of CLE-like effectors by CLVs should leads to more DEGs, so why there is no significant difference between the wild type and mutants? Is CLV1/2 not important or not the main receptors?

>We would like to clarify that this statement is referring to enriched GO terms in BCN up-/down-regulated genes, rather than the number of DEGs. No significant difference in enriched GO terms means that similar groups of genes are differentially regulated in syncytia formed in *clv* mutant and wild type plants. It is worth noting that samples collected from the *clv* triple mutant for RNA-seq have some syncytia, despite the lack of CLV1/2 and RPK2 receptors, whereas failed infection sites (which are more prevalent in the mutant) cannot be collected. This means that in the mutant, the nematode has circumvented disrupted CLE signaling pathways by activating alternative or parallel signaling pathways, and induced similar groups of DEGs for syncytium formation. It does not mean that CLV1, CLV2, and RPK2 are not important (the *clv* triple mutant forms less and smaller syncytia), but rather that BCN has a robust compensatory mechanism to circumvent the disruption of these receptors.

7. Line 214: Moreover, BCN infection may suppress the expression of downstream genes by secreting some effectors?

>We cannot exclude this possibility, but believe this is not the main reason for the similarity between BCN infected mutant and WT; it is more likely that it is caused by BCN effectors activating downstream signaling through other pathways.

8. Figure 2C and 2D: The same figures are presented in Figures S7 and S8. This is not standard. The same image should appear only once in the text.

>The panels for *ATHB8* expression were removed from Figure S7 and S8.

9. Lines 274-290: Moving this paragraph to the Discussion section may be better. Moreover, the logic and readability of this paragraph is not strong.

>This paragraph and corresponding panels were removed from Figure 2 (previous Fig 3F-J), as we think it does not provide conclusive information for the expression of *ATHB8* in the *clv* mutant.

10. Line 296: No difference of MIR165/166 expression has been reported previously, so why the authors chose MIR165/166? Removing this statement may be better.

>The statement is removed.

11. Lines 358-359: The numbers come from different experimental replicates are not suggested to do quantitative analysis.

>We consulted with the UGA Statistical Consulting Center. Per their suggestion, syncytium size from this experiment was re-analyzed using a Linear Mixed-effect Model, where the four biological replications were unified into one experiment, using the biological replicate as a blocking effect. In addition, syncytia measurements from the same well were averaged into a single mean value in the analysis. We have uploaded the statistical report for your review.

12. Line 440: Under the premise of functional redundancy, are other members doing the same to ATHB8? The authors may need to give a conclusion sentence that matches the Title in the manuscript.

>We edited the text to include other HD-ZIP III genes.

13. The discussion section is too descriptive. The authors should discuss the most significant results at different points. The relationship between ATHB8 and the CLE signaling pathway should be further discussed.

>The discussion section was revised to address these suggestions.

14. The citation format of References needs to be unified.

>Edited text to a unified citation format.

**Reviewer #3:**

**Part I - Summary**

Manuscript entitled ‘A Major Role of Class III HD-ZIPs in Promoting Sugar Beet Cyst Nematode Parasitism of Arabidopsis’ identified ATHB8, encoding a HD-ZIP III family transcription factor, as a downstream component of the CLE signaling pathway in syncytium formation, and knockdown of HD-ZIP III TFs by inducible over-expression of MIR165a in Arabidopsis dramatically reduced female development of the sugar beet cyst nematode. The authors obtained some promising experimental data.

**Part II – Major Issues: Key Experiments Required for Acceptance**

However, the results show that loss of function of ATHB8 and ATHB15/CAN does not affect BCN infection, and overexpression of ATHB8 does not increase susceptibility of Arabidopsis to BCN, meaning ATHB8 might not be associated with BCN susceptibility. If so, although auxin level and ATHB8 expression are elevated in neighboring cells of the developing syncytium, it is difficulty to conclude that HD-ZIP III TFs might function as a connecting point for CLE and auxin signaling pathways in promoting syncytium formation, possibly by inducing root cells into a quiescent status and priming them for initial syncytial cell establishment and/or subsequent cellular incorporation. I suggest authors continue to test the other HD-ZIP III TFs members which contribute(s) to BCN susceptibility in Arabidopsis. There are a lot of work to be done.

>Thank you for the suggestion. We tested BCN susceptibility of additional HD-ZIP III mutants, including *phb-13*, *phv-11*, *rev-6*, and the *phb-13 phv-11 cna-2* triple mutant (Fig S18). None of these mutants showed enhanced resistance to BCN infection, likely due to redundancy from other HD-ZIP III members. The ideal way to test the function of *ATHB8* in BCN infection is to test the *rev phb phv cna* quadruple mutant, which leaves *ATHB8* as the only functional HD-ZIP III gene. Nevertheless, this mutant is meristem-less in the shoot (Prigge et al., 2005) and has a complete loss of metaxylem in the root (Carlsbecker et al., 2010), thus is not suitable for BCN infection assays. Without the quadruple mutant, there is no definitive way to test if *ATHB8* is contributing to BCN susceptibility. Nevertheless, the fact that all five members need to be absent to completely disrupt xylem differentiation suggests that they are highly redundant in regulating vascular cell differentiation and all of them are likely to function in syncytium formation to some degree.

>We agree that it is hard to claim HD-ZIP III genes as a connecting point of CLE and auxin signaling. Thus, we have changed the text to read as “together with a CLE and auxin signaling network to promote syncytium formation”.

**Part III – Minor issues**

Lines 334-344, explained why knockdown of HD-ZIP III TFs by inducible over-expression of MIR165a in Arabidopsis, but did not check expression level of the five HD-ZIP III TFs.

>We did check the expression of HD-ZIP IIIs after *MIR165a* overexpression. Please see Figure 5A-B (previously Figure 6A-B) and line 316-324 (previously line 344-349) in the text.

There are too much RNA-Seq data analyses in this manuscript. Suggest to move Fig. 1A-D to supplement data, and Fig. 1 and Fig. 2 merge into one figure.

>Figures have been modified as suggested. Fig 1A was moved to Fig 2A. The rest of the Fig 1 panels were combined with Fig S3.

Fig. 3 shows the expression of ATHB8 was decreased in WT, and the mutants used GUS, the result may not be true; in addition with L289-290 ‘the GUS staining technique may not be sensitive enough to distinguish such a small difference in gene expression (Fig. 3A-J)’, I suggest authors add the GUS stain area for analysis, or conduct ATHB8 expression analysis at each stage after BCN infection by qPCR.

>We want to clarify that *ATHB8* expression is increased at the infection site compared to adjacent root tissue (or the same region of an uninfected root). It is the increased expression at the infection site that declines over time as the syncytium develops.

>We felt that quantification of GUS staining is not practical, as *ProATHB8::GUS* shows staining in all vascular tissues and the staining is not even throughout an uninfected root, making it difficult to apply a universal threshold to the GUS intensity.

>The goal of this experiment was to use an independent method to verify the qPCR result shown in Figure 1E, and doing qPCR at each stage after BCN infection stages would not serve this purpose.

>We removed Fig 3 (now Fig 2) panel F-J as they did not provide conclusive information for *ATHB8* expression in the *clv* triple mutant.

Does Fig.4 show the same root? The size are different, the bar needs to be added in each image.

>Yes, they are showing the same root. We felt that the figure is already too busy and since the scales are similar for each image that they can be shared across each panel. The main information we want to deliver here is the change of fluorescent signal intensity. We believe that sharing the same scale does not affect the conclusion made from this figure.

Fig. 6, The caption is wrong, it is for nematode development, but not for infection.

>Female development is a proxy for successful nematode infection (syncytium establishment).

Fig. 8, The image is not clear, I suggest authors add ‘Bright Field’ single channel.

>We added bright field to Figure 7A (previously Figure 8A) and Fig S22A (previously Figure S19A).

Supplemental figures, all ‘um’ are replaced with ‘μm’.

>We changed all ‘um’ to ‘μm’.

**Reviewer #4:**

**Part I – Summary**

In this work, the authors investigated the potential function of Class III HD-ZIP TF genes in promoting sugar beet cyst nematode (SBCN) infection of Arabidopsis roots. Based on the evidence obtained, they suggest that Class III HD-ZIP TF genes play a major role for efficient parasitism of Arabidopsis by SBCN. The major strength is that the authors endeavored to overcome functional redundancy of HD-ZIP TF genes during their regulation of SBCN infection. The weakness of the work is that silencing the expression of Class III HD-ZIP TF genes by inducible expression of MIR165a had highly variable effects on SBCN infection of Arabidopsis, which do not support the claim that Class III HD-ZIP TF genes play “a major role” in promoting SBCN parasitism of Arabidopsis.

**Part II – Major Issues: Key Experiments Required for Acceptance**

1) The authors used the number of SBCN females per plant at the lates stage of infection as a parameter to assess the effects of silencing HD-ZIP TF genes, and they conclude that silencing HD-ZIP TF genes impairs syncytium formation. However, they should examine SBCN infection sites per plant at the early and middle stages of the infection, e.g., 1, 3, 4, 6, 12 days after inoculation, to reveal if lowering HD-ZIP TF gene may indeed impair the initiation and/or early development of syncytium.

>Adult female counts at 14 dpi is a common metric to evaluate BCN infection in the field. It would be ideal to check syncytium formation at earlier stages, but there is no reliable biomarker or consistent phenotype to do so due to the un-synchronized nature and high variability of BCN infection. We concluded that HD-ZIP III functions in the early stages of BCN infection because HD-ZIP III genes are only suppressed at the early days of induction in both *Pro35S::XVE>>miR165a* and *ProCRE1::XVE>>miR165a* lines (Fig 5A-B), and number of females at 14 dpi is a proxy for successful nematode infection (syncytium formation).

2) The authors suggest that upregulation of HD-ZIP TF genes by SBCN infection may facilitate early syncytium formation/development possibly by inducing root cells into a quiescent status. To support this proposition, the authors are advised to use suitable biomarkers to check if cellular quiescence may indeed occur in the roots at the early or middle stages of SBCN infection.

>All biomarkers for cellular quiescence we found in the literature are for the quiescence center at the root apical meristem, which is very different from quiescent cells at the vasculature. In the Smetana 2019 paper, the authors showed that high expression of *ATHB8* is sufficient to induce cells into a quiescent status, while HD-ZIP III suppression forced quiescent cells to re-enter mitosis. These results suggest that high auxin and *ATHB8* levels around the syncytial cell likely promote these cells into quiescent status.

**Part III – Minor issues**

Because syncytium size was not significantly decreased by silencing HD-ZIP TF genes, it is difficult to understand the authors’ suggestion that HD-ZIP TF genes may be involved in cellular incorporation during syncytium formation in the roots with HD-ZIP TF gene expression lowered.

>We re-did the statistical analysis using a linear mixed-effect model suggested by the UGA statistical consulting center and have uploaded the analysis report for your review. The new analysis shows that syncytium size in both inducible lines is significantly reduced compared to that of controls (Fig 5E).

>We recognize that the extent of the syncytium size reduction is small. There are several factors to consider in interpreting this result, 1). HD-ZIP III genes are not completely knocked out. The remaining HD-ZIP III expression could be sufficient for some nematodes to successfully establish a syncytium that expands relatively normally later on as expression of HD-ZIP III genes recovers. 2). Suppression of HD-ZIP III genes is only temporary after estradiol induction. As HD-ZIP III expression recovers some BCN could start a new infection and to catch up on syncytium expansion. 3). In Arabidopsis, the syncytium usually reaches its final size 10 days after establishment. Measuring syncytium size at 15 dpi allows syncytia that were initiated late extra time to catch up in expansion. However, measuring syncytium size early could lead to a misleading result, as relatively more syncytia are expected to be established late due to the suppression of HD-ZIP III genes in inducible lines. Measuring syncytia size early cannot separate the effect of delayed syncytium establishment and reduced syncytium expansion. 4), In addition, we also need to consider that syncytia that failed to expand sufficiently to make contact with xylem and phloem at early stages will degenerate, which results in reduced female development. But these syncytia cannot be captured for syncytium size measurement.

>Despite all of these factors, we still observed significant reduction in syncytium size, thus we conclude that HD-ZIP III genes are involved in cellular incorporation during syncytium expansion.
